# Telomeres, telomerase, and cancer: mechanisms, biomarkers, and therapeutics

**DOI:** 10.1186/s40164-025-00597-9

**Published:** 2025-01-27

**Authors:** Songting Shou, Ayidana Maolan, Di Zhang, Xiaochen Jiang, Fudong Liu, Yi Li, Xiyuan Zhang, En Geer, Zhenqing Pu, Baojin Hua, Qiujun Guo, Xing Zhang, Bo Pang

**Affiliations:** Guang’anmen Hospital, China Academy of Chinese Medical Sciences, Beijing, China

**Keywords:** Telomeres, Telomere shortening, Cancer

## Abstract

Telomeres and telomerase play crucial roles in the initiation and progression of cancer. As biomarkers, they aid in distinguishing benign from malignant tissues. Despite the promising therapeutic potential of targeting telomeres and telomerase for therapy, translating this concept from the laboratory to the clinic remains challenging. Many candidate drugs remain in the experimental stage, with only a few advancing to clinical trials. This review explores the relationship between telomeres, telomerase, and cancer, synthesizing their roles as biomarkers and reviewing the outcomes of completed trials. We propose that changes in telomere length and telomerase activity can be used to stratify cancer stages. Furthermore, we suggest that differential expression of telomere and telomerase components at the subcellular level holds promise as a biomarker. From a therapeutic standpoint, combining telomerase-targeted therapies with drugs that mitigate the adverse effects of telomerase inhibition may offer a viable strategy.

## Introduction

Cancer's global burden continues to mount, imposing significant strain on nations worldwide. In certain regions, it has even eclipsed stroke and coronary heart disease to become the foremost cause of premature mortality [1]. With the advancement of research, there is an increasing focus on early detection and timely intervention in cancer care, strategies that have been shown to markedly improve patient survival outcomes [2]. This principle holds true across all cancer types. Nonetheless, realizing this objective remains formidable due to the persistent array of challenges. Achieving success in this endeavor necessitates a profound understanding of cancer biology, thorough assessment of individual risk profiles, identification of optimal cancer biomarkers, development of robust detection strategies, and meticulous evaluation of these methodologies [2]. Therefore, it is essential to identify an entry point to discover a substance that is intricately linked to the initiation and progression of cancer and can also function as a biomarker. Detection methods centered on this substance could be employed to evaluate patient cancer risk cancer risk. Targeting cancer cells therapeutically necessitates the fulfillment of two fundamental conditions [3]. The first condition is that the target must be specific and able to differentiate cancer cells from normal cells. The second condition is that the target must be critical for the survival of cancer cells, such that its inhibition reduces their viability. Telomeres and telomerase may indeed fulfill these criteria.

**Fig. 1:**
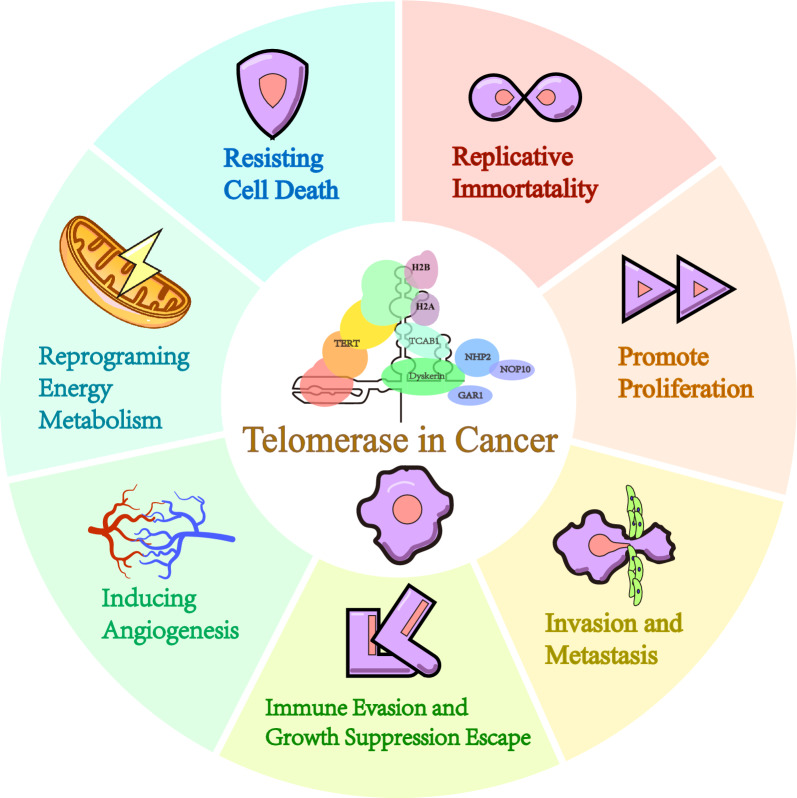
Telomerase and Its Role in Cancer Progression. Telomerase enables the immortalization of cancer cells, while also promoting their proliferation, metastasis, invasion, and immune evasion. Additionally, it facilitates angiogenesis, regulates metabolic processes, and safeguards cancer cells from growth inhibition and death

During the initiation and progression of tumors, two principal barriers are cellular senescence and crisis [4]. Cellular checkpoints activate apoptotic pathways and induce cell cycle arrest in cells experiencing senescence or crisis. This regulatory mechanism preserves a relatively healthy and stable cellular milieu [5, 6]. Tumor cells progress through a series of genetic and epigenetic modifications, enabling them to overcome these barriers and evade the homeostatic controls typically enforced by tissue structure and function. A pivotal mechanism driving this oncogenic escape is the shortening of telomeres accompanied by the activation of telomerase [7, 8]. Furthermore, telomerase plays a multifaceted role in the pathogenesis of cancer, encompassing not only the promotion of tumor cell proliferation and invasion but also contributing to immune evasion and other critical processes that facilitate tumorigenesis [9] (Fig. 1). Moreover, telomerase can serve as a tumor marker due to it’s overexpressed in approximately 85% of tumor cells [10]. On the other hand, telomere shortening fosters genomic instability, subsequently precipitating chromosomal aberrations and heightening the risk of carcinogenesis [11]. Telomere length may additionally function as a biomarker indicative of cancer risk [12]. Some studies suggest that when telomerase activity (TA) in cancer cells declines to undetectable levels, the tumor may cease growth or undergo spontaneous regression [13–15]. It is noteworthy that telomeres possess an alternative elongation mechanism that bypasses the requirement for telomerase, termed alternative lengthening of telomeres (ALT). The occurrence of ALT, however, remains relatively rare. In this review, we summarize cutting-edge and typical research articles related to telomeres and telomerase. We trace their roles in cancer development by delineating their functions at various stages. Biomarkers linked to telomeres, telomerase, and cancer are introduced, and the potential for targeting telomeres and telomerase in future cancer therapies is explored.

## The structure of telomeres and telomerase

### Telomeres and telomerase ensure the stability of growth and development

**Fig. 2:**
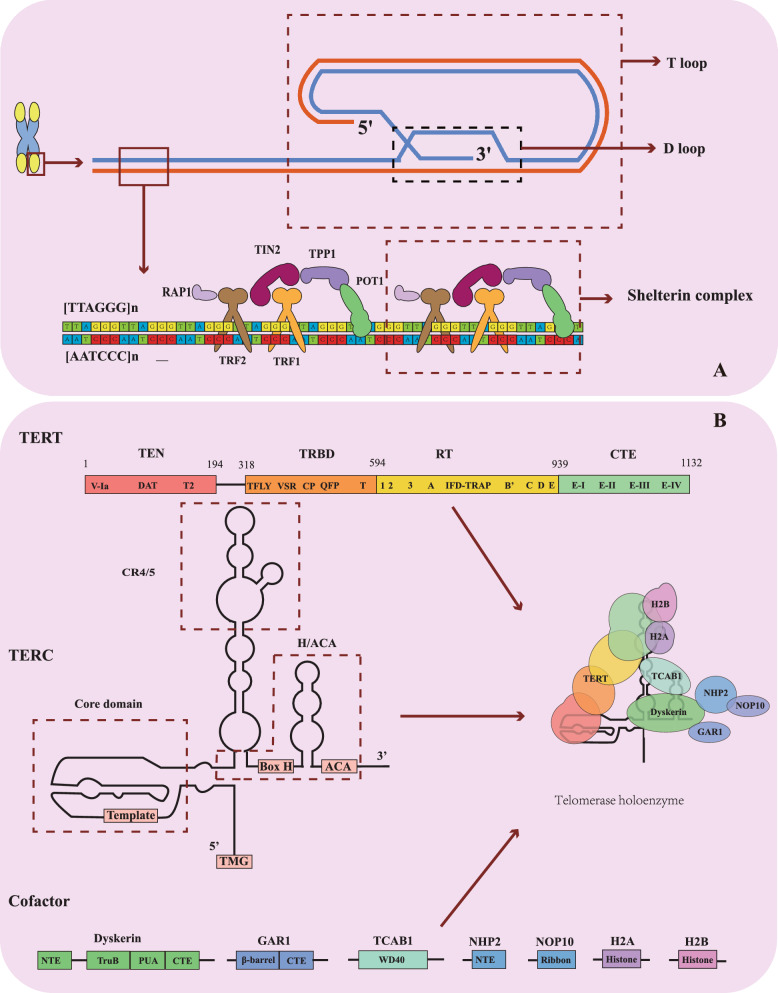
The Structure of Telomeres and Telomerase. **A** Structure of Telomeres and Shelterin complex. POT1, protection of telomeres 1. RAP1, repressor/activator protein 1. TIN2, TERF1-interacting nuclear factor 2. TPP1, telomere protection protein 1. TRF1, telomeric repeat binding factor 1. TRF2, telomeric repeat binding factor 2. **B** Structure of Telomerase holoenzyme. TEN, Telomerase Essential N-terminal. TRBD, Telomerase RNA Binding Domain; RT, reverse transcriptase domain and CTE, C-Terminal Extension domain. PK/T, pseudoknot/template. CR4/5, conserved regions 4 and 5. GAR1, nucleolar protein family A. member 1. NHP2, nucleolar protein family A, member 2. NOP10, nucleolar protein 10. TCAB1, telomerase Cajal body protein 1. H2A, Histone 2A. H2B, Histone 2B. TERT and the H2A-H2B dimer constitute the catalytic core in conjunction with the PK/T and CR4/5. The remaining subunits interact with the H/ACA domain, forming the H/ACA lobe. Box H, Hairpin Box. ACA, “ACA” Box. TMG, Trimethylguanosine Cap.

Telomeres consist of telomeric DNA and the shelterin complex [16, 17] (Fig. 2A). The telomeric DNA features a repetitive, double-stranded sequence, TTAGGG in mammals, extending to lengths of thousands to even tens of thousands of base pairs [16]. At the terminal region, the strands are asymmetrical in length, with the 3′ end forming the longer strand and the 5′ end the shorter [18]. These strands wrap around to form D-loop and T-loop structures [18]. The shelterin complex consists six protein subunits: telomeric repeat-binding factors 1(TRF1), TRF2, protection of telomeres 1 (POT1), the TRF1-interacting protein 2(TIN2), POT1-TIN2 organizing protein (TPP1), and repressor/activator protein 1 (RAP1). This complex binds to the telomeric DNA, preserving telomere integrity and stability and thereby preventing telomeric DNA damage [17, 19, 20].

The telomerase is composed of three essential components: telomerase RNA component (TERC), Telomerase reverse transcriptase (TERT), and cofactors [21, 22] (Fig. 2B). TERC provides the template for reverse transcription, TERT performs the reverse transcription, and auxiliary factors support TERT in this process. Together, these components synthesize telomeres in a coordinated manner. Structurally, TERT organized into four domains: the Telomerase essential n-terminal (TEN) domain, Telomerase RNA binding domain (TRBD), reverse transcriptase (RT) domain, and C-Terminal extension (CTE) domain [17, 22–24]. Telomerase is restricted in its expression to certain cells in the human body, thereby enabling these cells to sustain long-term proliferation and survival while also reducing the risk of cancer [25]. The regulation of TA in the human is primarily achieved by controlling the transcription of TERT [25]. Human TERT (hTERT) is generally expressed in fetal tissues, but its expression becomes restricted after birth [26]. Consequently, TERT expression is typically observed in human embryonic stem cells, neural stem cells, bone marrow progenitor cells, and malignancy [27–29].

### Telomeres and telomerase have shown abnormalities in precancerous lesions

From the perspective of telomeres and telomerase, current research suggests that the onset of cancer is not an abrupt event; rather, the human body exhibits certain diseases in the early-stage diseases may predispose certain tissues to cancer development. For instance, in precancerous prostate lesions, such as high-grade prostatic intraepithelial neoplasia (HGPIN), telomeres in glandular epithelial cells are significantly shortened [30]. In studies of pancreatic cancer, progressive telomere shortening in intraductal papillary mucinous neoplasm (IPMN) primarily occurs during the early stages of IPMN carcinogenesis, preceding telomerase activation, which becomes pronounced only as the disease progresses. Similarly, in the low-grade dysplastic nodule preceding hepatocellular carcinoma (HCC), hepatocytes exhibit both telomere shortening and telomerase activation. Significant changes in TA and telomere length are observed during the progression from low-grade dysplastic neuroepithelial (LGDN) to high-grade dysplastic neuroepithelial (HGDN) nodules [31]. These findings indicate that telomere shortening and subsequent telomerase activation occur in premalignant tissue cells, foreshadowing cancer development. In gastric cancer research, it was observed that telomere shortening in gastric epithelial cells begins early, following Helicobacter pylori infection [32]. Helicobacter pylori continuously damages the gastric mucosa, accelerating its regeneration whole the intestinal metaplasia induced by Helicobacter pylori also displays high proliferative activity [32–34]. These two factors together increase cellular division rates, ultimately resulting in telomere shortening. Following telomere shortening in gastric cancer epithelial cells, there was an emergence of hTERT expression in the cells, suggesting a potential causal relationship between the two events, where telomere shortening leads to hTERT expression and subsequently activates telomerase [32, 34]. These hTERT-positive cells are all located at the base of the glands, where the epithelial stem cells reside [32, 35]. Thus, enhancement of hTERT expression may initially occur primarily in stem cells that inherently possess TA. Moreover, studies indicate that in certain diseases, particularly those associated with an elevated risk of cancer, the early stages are characterized by significant telomere shortening, leading to genomic instability. As mutations accumulate, telomere attrition may persist until the development of precancerous lesions, subsequently triggering telomerase activation and facilitating the transition to malignancy. This early telomere shortening is typically transient. For instance, telomere attrition occurs early in colon carcinogenesis, with telomere shortening observed in low-grade dysplasia (LGD). This attrition reaches its peaks in high-grade dysplasia (HGD), however, as tumors acquire full invasive potential, telomere length may be restored to levels comparable to those of normal tissue [36]. Additionally, some studies have reported TERT promoter mutations in early precancerous lesions of various cancers, including HCC, bladder cancer, cutaneous melanoma, and squamous cell carcinoma [37–40]. The progression from precancerous lesions to cancer can potentially be categorized into distinct phases based on telomeres and telomerase activity: the telomere shortening phase, telomerase activation phase, and telomere restoration phase. These phases may represent the increased risk of cancer, carcinogenesis, and cancer invasion, respectively.

## Abnormal telomeres increase the risk of cancer

### Telomere shortening leads to abnormal anti-cancer function

**Fig. 3:**
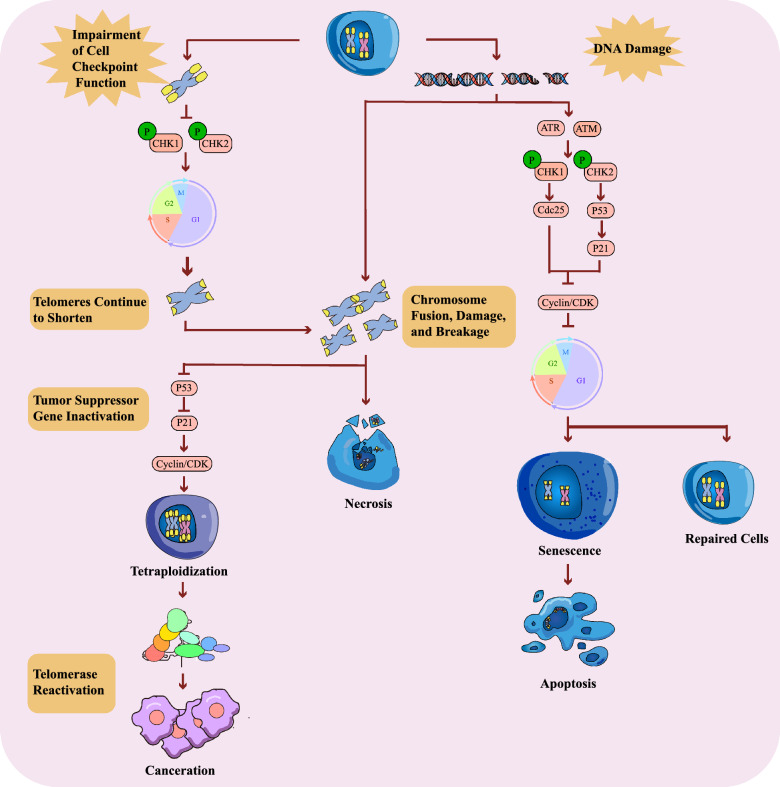
Telomere Shortening and Cellular Checkpoints. Under the influence of aging and disease-related factors, telomere shortening results in an increase in chromosomal instability. If the telomere checkpoint remains functional, cells will cease further replication once telomeres reach a critical length. When DNA damage checkpoints are intact, single-strand or double-strand breaks occurring during replication activate repair mechanisms, including the ATM and ATR signaling pathways. These pathways exert their effects by inhibiting the activity of the Cyclin/CDK complexes, thereby halting cell cycle progression and allowing sufficient time for DNA repair. Should repair fail, the cell will undergo either senescence or apoptosis. However, in cases where checkpoint mechanisms are impaired, cells are unable to exit the cell cycle, leading to further telomere shortening and gradual accumulation of chromosomal abnormalities. During this process, chromosomal damage or fusion events may trigger cell death. If cell cycle checkpoints are inactivated and tumor suppressor genes such as p53 are compromised, abnormal cell division may occur, thereby increasing the likelihood of tetraploidy and exacerbating chromosomal instability. At this juncture, activation of telomerase bypasses the restrictions imposed by aging and cell death, enabling the accumulation of mutations within the immortalized cell, which eventually evolves into malignant tumor cells.

Telomere systems exhibit substantial variability across mammalian species. In mice, telomeres typically range from 40–60 kilobases in length, whereas in humans, they measure approximately 10 kilobases. Most murine cells express telomerase activity, which is largely undetectable in adult human cells. This distinction elucidates why mouse fibroblasts often undergo spontaneous immortalization, in contrast to human fibroblasts [41]. Epidemiological studies indicate that around 30% of laboratory rodents develop cancer by the end of their 2–3 year lifespan, a statistic mirrored by the roughly 30% of humans who develop cancer by age 70–80 [41, 42]. Furthermore, the genetic tractability of murine models facilitates the development of animal models that closely resemble human biology [41]. Consequently, murine models remain indispensable for investigating the interplay between telomeres, telomerase, and cancer [41]. In the fetal period, telomeres achieve their initial length of approximately 15–20 kilobases (kb) due to the action of telomerase [26]. Postnatally, a three-dimensional chromatin structure, characterized by telomere position effects over long distances (TPE-OLD), typically silences the TERT gene in most somatic tissues, thereby halting further telomere elongation [43, 44]. Studies have demonstrated that when telomerase-deficient mice are bred with various tumor model mice, the p53 pathway collaborates with critically short telomeres to restrict tumor development [43, 44]. Telomere fusions, initiated by critically shortened telomeres, can disrupt oncogenic receptor loci of T-cell receptor on chromosome 14 [45]. Additionally, translocations were detected at these loci in pre-malignant thymocytes, phenomena which both contribute to the attenuation of tumor formation [45]. The identification of recurrent activating mutations in the TERT promoter across several types of human cancers, along with the widespread mutations in p53 observed in cancers, may provide some evidence suggesting that the reactivation of telomerase through heightened TERT expression enables cancer cells to bypass the telomere-p53 checkpoint, thereby contributing to cancer progression [46] (Fig. 3). The p53 tumor suppressor protein serves as the primary mediator of the DNA damage response, inducing apoptosis and senescence. In cellular replication and senescence, these processes involve multiple pathways, many of which are linked to mitochondria and the mitochondrial genome (mtDNA) [47]. Telomere dysfunction during aging exerts detrimental effects on mitochondria, partially mediated by p53 [48]. Both p53 and TERT regulate their functions via reactive oxygen species (ROS) signaling, where physiologically low levels of ROS confer protective effects. In contrast, elevated ROS levels can directly or indirectly induce mtDNA damage, partially mediated by interactions involving p53, TERT, and microRNA (miRNA) [47]. Shortened telomeres can effectively redirect the p53-mediated tumor suppressor response towards cellular senescence [49]. However, mice with short telomeres are more susceptible to cancer development once p53 function is compromised [49]. As telomeres progressively shorten over time and chromatin structure becomes less compact, this silencing effect is diminished, potentially enabling the reactivation of TA under certain conditions [43, 44]. Notably, shortened telomeres increase susceptibility to DNA damage [50]. When DNA damage occurs, it often leads to the inactivation of tumor suppressor pathways, particularly those involving p53 and Rb proteins [51, 52]. These pathways are closely related to cellular checkpoints [53]. The mechanism underlying the transition from telomere shortening to carcinogenesis involves the cellular response to telomere attrition. In response to both external and internal stimuli that jeopardize cellular genetic stability, telomeres elicit an expedited response. Perturbations such as telomeric chromatin structural disruption, DNA double-strand breaks, replicative stress, and ectopic recombination can trigger telomere-related checkpoint signaling. Consequently, this activates cell-cycle arrest and programmed cell death, thereby preserving genomic integrity [5]. This response is contingent upon the integrity of cellular checkpoint functions. In most instances, as telomeres shorten, cells undergo senescence or crisis, leading to cell death or growth arrest. This mechanism can be defined as a telomere checkpoint, which functions as one of the anticancer defenses in multicellular organisms [5]. However, under certain circumstances, individual cells with compromised checkpoint functions may continue to shorten their telomeres until reaching a state of replicative crisis [54, 55]. Among these crisis-prone cells, a subset may further lose checkpoint function entirely, thus escaping replicative crisis once again and ultimately transforming into neoplastic cells [54, 55]. Telomere shortening and subsequent dysfunction serve as a tumor-suppressive mechanism through the activation of cellular checkpoints.

However, when checkpoint function is compromised, telomere dysfunction can lead to genomic instability, promoting the transformation of cells into neoplastic cells, which are also referred to as pre-neoplastic cells during this stage [46]. At this point, if telomerase is activated, it initiates telomere repair, thereby enabling cancer cells to evade the activation of cellular checkpoints and permitting them to proliferate with a stable genome, ultimately forming tumors with invasive and metastatic capabilities [56]. This sequence of events ultimately facilitates the unchecked proliferation of tumor cells. Numerous studies have also demonstrated that the primary cause of cancer cell proliferation is the impairment of the cell's ability to exit the cell cycle [57]. Cancer cells employ diverse mechanisms to reactivate telomerase, sustaining telomere length and enabling unchecked proliferation. Restoring endogenous p53 expression resulted in the regression of lymphomas and sarcomas in mice without affecting other normal tissues [58]. Upon reactivation of p53, a strong inflammatory response was initiated, subsequently disrupting both tumor cells and the neovascular system. Inflammatory cytokines produced by macrophages, neutrophils, and natural killer cells were upregulated in animal models of tumors shortly after p53 reactivation but not in vitro experiments. This suggests that p53 may activates innate immune system functions, leading to the secretion of substances by the innate immune system to infiltrate and eliminate tumors [59].

### Abnormal activation of shelterin complex in cancer

Telomeres are not only necessary for the continuous replication of normal cells, but also for the continuous replication of cancer cells. The integrity of telomeres relies on the protection of shelterin proteins. Cancer cells must evade telomere DNA damage, a consequence of their abnormal oxidative metabolism, to ensure continuous replication and survival [60]. In cancer, changes in various parts of the shelterin complex can lead to telomere dysfunction [61].

The primary significance of TRF1 and TRF2 lies in their regulation of telomere length. TRF1 and TRF2 are responsible for assembling several other shelterin proteins, so their functions play an important role in protecting telomeres [62]. By modulating telomere length, these proteins are also able to influence a variety of cellular functions in cancer cells. TRF1 and TRF2 are highly expressed in both renal cell carcinoma and gastric cancer [63, 64]. Although studies have shown that an increase in TRF1 and TRF2 leads to telomere shortening and a decrease in TERT [65, 66]. TRF1 was positively correlated with telomere length in pancreatic cancer and breast cancer [67]. This suggests that these three genes may have more complex roles in the regulation of telomere length. A deficiency in TRF1 can result in accumulation of telomere DNA damage, leading to genetic instability, cell apoptosis, proliferation issues, and mitotic catastrophes within cancer cells [68]. In radio-resistant breast cancer cells, the expression of TRF1 and POTP increased [67]. Interestingly, prolonged absence of TRF1 does not seem to impact the health of adult organisms, which has sparked interest in developing related therapeutic agents to target cancer [68]. TRF2 also exhibits similar functions in many cancers. When TRF2 is knocked down, it leads to inhibition of growth, proliferation, and migration in gastric cancer cells, accompanied by significant telomere dysfunction. This is further associated with cell apoptosis, autophagic cell death, and ferroptosis [69].The expression of TRF2 can make tumor cells resistant in glioblastoma and gastric cancer [40, 70]. NIMA-related kinases 7(Nek7) not only protects Telomeres from oxidative DNA damage by photoshoresis and stability of TRF1, but also increases its expression in cancer [60]. Studies in rectal cancer have shown that Specificity Protein 1(Sp1) is involved in the upregulation of TRF2 expression, suggesting that SP1 may contribute to the stable replication of cancer cells through this mechanism, thereby potentially facilitating cancer progression [71]. SP1 not only maintains basal transcription but also possesses functions in inducing and inhibiting the transcription of cellular genes [72–74]. In normal cells, SP1 participates in proliferation, differentiation, the DNA damage response, apoptosis, and senescence, and it may also contribute to whole-chromosome stability during mitosis [75]. SP1 can also interact with p53 at the TERT promoter, preventing TERT expression [76, 77]. TRF2 facilitates the folding and protection of telomeric DNA. Consequently, elevated levels of TRF2 in tumors may aid in maintaining telomeres within cancer cells. Additionally, TRF2 may assist tumor cells in evading immune surveillance, since inhibition of TRF2 has been shown to promote the activation of NK cells [78].From the perspective of telomere biology, NEK7 and SP1 may also emerge as potential targets for future cancer therapies.

TIN2 must associate with TRF1 to facilitate its recruitment to telomeric regions, whereupon it interacts with TRF1, TRF2, and TPP1, serving a pivotal role in the stabilization of the shelterin complex [79, 80]. The absence of TIN2 disrupts the functional integrity of TRF2, POT1, and TPP1, and subsequently triggers the activation of the ATM and ATR-mediated DNA damage response pathways [18, 80]. When TIN2 undergoes mutation, it can result in two divergent outcomes [81]. Firstly, the mutation may lead to cancer by facilitating excessive telomere elongation. Conversely, it can also cause bone marrow failure syndromes by accelerating telomere shortening. While it is established that critically short telomeres can predispose cells to cancer, emerging evidence also indicates that unusually long telomeres confer an increased risk of carcinogenesis [82].

In humans, the recruitment of Rap1 is dependent on TRF2 and lacks the ability to bind directly to telomeric DNA sequences [83]. Rap1 plays a role in inhibiting homologous directed repair (HDR), a process that can alter telomere length and promote chromosomal fusion [84, 85].In the lacking of RAP1, the induction of telomeric repeat-containing RNA (TERRA) transcription ensues [86]. TERRA, transcribed from telomeres in vertebrates, is an RNA molecule containing telomeric repeat sequences. It belongs to a class of heterogeneous RNAs, ranging in size from 100 base pairs to more than 9 kilobases [87]. The deficiency of RAP1 leads to the binding of TERRA to TERT and the telomerase RNA component (TERC), thereby hindering the interaction between TERT and TERC [86]. As a consequence, the catalytic activity of telomerase, which is crucial for telomere maintenance, is significantly reduced [86]. The expression of RAP1 can influence the activation of NF-κB, and in turn, NF-κB can positively regulate the level of RAP1 [88]. In breast cancer cells, both RAP1 and NF-κB levels are often elevated. Knockdown of either RAP1 or NF-κB can sensitize breast cancer cells to apoptosis induced by doxorubicin [88]. In NSCLC cells characterized by high RAP1 expression, the initial activation of nuclear factor NF-κB is observed. This activation subsequently induces the upregulation of B-cell lymphoma 2 (BCL-2), a key anti-apoptotic protein. The resultant overexpression of BCL-2 confers resistance to cisplatin, a commonly used chemotherapeutic agent [89]. These studies suggest that elevated expression of RAP1 may indicate telomere elongation and an increase in the number of shelterin complexes on telomeres, thereby protecting chromosomal stability and preventing apoptosis. Conversely, decreased RAP1 expression may indicates a failure of the shelterin complex to protect telomeres, leading to increased susceptibility to apoptosis induction.

TPP1 facilitates the recruitment of POT1, directing its localization to the telomere [90, 91], and serves as a crucial protein for tethering telomerase to the telomere [85]. TPP1 and TIN2 share functional similarities as components of the shelterin complex. Studies on esophageal cancer models demonstrate that TPP1 exerts its effects through the ATM/ATR-p53 signaling pathway, with two notable impacts: it decreases the sensitivity of cancer cells to cisplatin and reduces DNA damage, possibly by stabilizing telomere structure or enhancing DNA repair mechanisms [92]. In research on cervical cancer, it has also been found that high expression of TPP1 is associated with cervical intraepithelial neoplasia (CIN) and a high risk of cervical cancer [93]. This phenomenon may be attributable to the telomere elongation facilitated by the upregulation of TPP1. Chun-On P and colleagues, in their melanoma experiments, elucidated that promoter mutations in the TPP1 gene leading to its overexpression do not singularly impact telomere length [94]. Yet, in conjunction with TERT overexpression driven by promoter mutations in the TERT gene, a synergistic effect manifests, resulting in telomeres that are notably longer compared to those observed under conditions of TERT overexpression alone [94].

POT1 is essential for maintaining telomere integrity; it not only binds to DNA substrates but also interacts with TERT in a more dynamic manner than TPP1, thereby regulating telomere dynamics [95, 96]. The human POT1 protein utilizes its two N-terminal oligonucleotide/oligosaccharide-binding (OB) fold domains, known as OB1 and OB2, to identify and adhere to single-stranded telomeric DNA [97]. Across a diverse range of cancer, including melanoma, cardiac hemangioma, glioma, and colorectal cancer, mutations in the POT1 gene have been implicated in the deregulation of telomere maintenance, manifesting as telomere elongation and enhanced telomere fragility [98–101]. These genetic alterations disrupt the normal function of POT1, a critical component of the shelterin complex, thereby contributing to genomic instability and the progression of cancer through aberrant telomere dynamics. And these mutations are often located in OB1 and OB2, with a small number of mutations coming from the C-terminal region of POT1 [98–102].

## The reactivation of telomerase leads to cancer

### High expression of TERT is key to telomerase reactivation

The activity of human telomerase is linked to the expression of TERT, which is regulated by the TERT promoter and associated transcription factors [103]. Consequently, aberrant expression of the TERT promoter or dysregulation of transcription factors in cancer can result in altered TERT expression, leading to abnormal TA in cells.

**Fig. 4:**
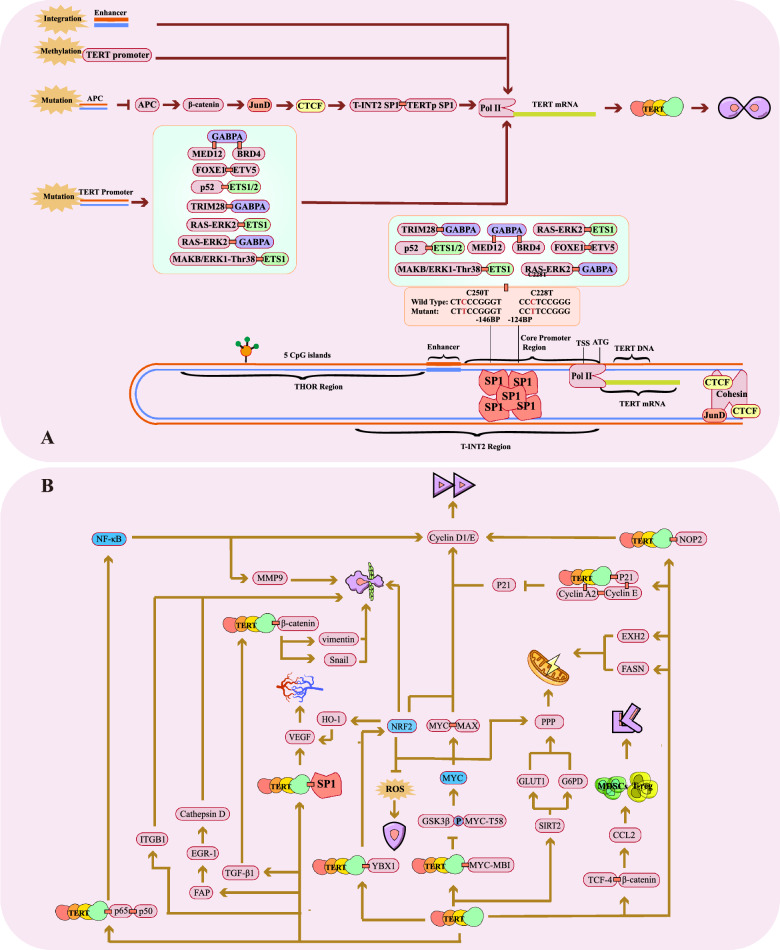
Mechanisms of telomerase activation and TERT-driven cancer progression. **A** Telomerase activation through TERT expression occurs via four primary mechanisms: enhancer insertion, promoter methylation, chromosomal abnormalities, and promoter mutations. The insertion of enhancers and methylation of the promoter region alleviate the suppression of the TERT promoter, thereby initiating its expression. Chromosomal abnormalities, often resulting from mutations in genes such as APC, lead to the accumulation of JunD, which relaxes the chromatin structure. This facilitates long-range chromatin interactions mediated by Sp1, driving the activation of the TERT promoter and subsequent TERT expression. Promoter mutations involve alterations in the promoter region across various cancers, where specific mutation sites interact with distinct complexes to ultimately drive TERT expression. B, TERT can exert numerous functions beyond telomere maintenance within the cell, and through these functions, it facilitates the initiation and progression of cancer. As depicted in the figure, the activation of NF-κB, NRF2, and MYC (represented in blue) can, in turn, promote the expression of TERT.

The upregulation of TERT expression involves a diverse array of mechanisms. To date, studies have identified four principal pathways: promoter mutations, promoter methylation, the insertion of enhancer elements into the promoter region, and alterations in chromatin architecture (Fig. 4A) [24, 25] (Fig. 4). The most prevalent mechanism is promoter methylation (53%) [104]. In normal human cells, promoter methylation typically disrupts the binding of transcription factor to their respective sites, resulting in the silencing of associated genes. This process plays a critical role in regulating organ and tissue functionality during development [105]. In cancer cells, a TERT hypermethylated oncological region (THOR) is located distal of the transcription start site (TSS). The region comprising 52 high-frequency repetitive DNA segment containing CG sequences, commonly referred to as CpG islands [106, 107]. When unmethylated, this segment inhibits TERT transcription; However, upon methylation, it loses this inhibitory capacity after methylation, leading to TERT overexpression. The second most common mechanism involves TERT promoter mutations (31%) [104]. These mutations emerge following telomere shortening, extending cellular survival while telomeres continue to shorten until a critical threshold is reached, triggering genomic instability and subsequently increasing TERT promoter activity [29]. Co-occurrence of TERT promoter mutation with mutations in other genes such as BRAF, FGFR3, and IDH is associated with poorer prognoses. In cancers such as papillary thyroid carcinoma, bladder cancer, and glioma, such mutations correlate with outcomes higher recurrence rates and reduced survival outcomes [108–112]. Interestingly, certain cancers, including lung and prostate cancer exhibit few TERT promoter mutations. Nonetheless, TA, persists in these cancers, suggesting the existence of additional, yet-undiscovered mechanisms underlying TERT upregulation [113–115]. TERT promoter mutations are associated with patients’ advanced age and environmental exposures such as ultraviolet radiation and chemical carcinogens [116–118]. Frequent observed mutations in TERT promoters include C > T transitions at -124 bp (C228T) and -146 bp (C250T) upstream of the TSS [116, 119]. The TERT promoter contains numerous binding sites for transcription factors, including c-MYC, SP1, NF-κB, members of the STAT protein family, AP-2, and GSC, all of which contribute to TERT activation [113]. Moreover, certain mutations in the TERT promoter create novel sequences that serve as consensus binding sites for E-twenty-six (ETS) transcription factors, further driving TERT expression [120, 121]. But this may not happen in all cancers. Mutations in the TERT promoter are observed at high frequencies in tumors characterized by lower proliferative potential, including melanoma and HCC, but are infrequent or virtually absent in highly proliferative cancers, such as breast and testicular cancer [38, 113, 119, 122]. In addition, telomere dysfunction-induced DNA damage has been implicated in rare instances, where it heightens the likelihood of chromosomal rearrangement, thereby facilitating the emergence of enhancers or gene amplification in proximity to the TERT locus [123, 124]. DNA damage may activate the RAS oncoprotein, thereby leading to upregulation of TA [125]. The specific mechanisms underlying this process require further investigation. However, the upregulation of TA in this context may represent a physiological protective mechanism aimed at facilitating DNA repair and enhancing cellular survival [126]. Chromosomal alterations have been shown to involve a well-characterized mechanism, exemplified by mutations in the APC gene that result in the upregulation of β-catenin [127]. This upregulation drives JunD-mediated enrichment of CTCF at specific chromosomal loci, inducing changes in chromatin architecture. Such structural reorganization ultimately facilitates long-range chromatin interactions, culminating in the reactivation of the TERT promoter. In HCC, the virus exerts a pivotal role by mediating the chimeric fusions of the C-terminus region of its HBx gene with the human TERT promoter, resulting in elevated expression of TERT mRNA. This aberrant structure configuration is driven by ELF4, which binds to the fused HBV EnhI element at the TERT promoter [128].

### High expression of TERC promotes the occurrence and development of cancer

Earlier research predominantly focused on TERT, while TERC's role was initially less emphasized. Recent studies, however, have revealed that it frequently undergoes amplification in numerous cancers, highlighting its critical involvement in telomerase function and cancer progression [129–131]. TERC is an essential component of TERT's tumor promoting effect. In the absence of TERC involvement, overexpression of TERT cannot upregulate TA and instead may have an inhibitory effect on tumors [132]. In all stages of prostate cancer, including precancerous lesions, TERC consistently exhibits overexpression under the regulatory influence of MYC [129]. This overexpressed TERC fosters proliferation of tumor cells and promotes tumor growth [129]. The overexpression of TERC in cancer may be related to the lower degree of methylation of the TERC promoter, which also indicates the development of the disease [133]. In addition, TERC may exert its effect through a certain part [134]. A 23-nucleotide RNA, designated TERC-sRNA, enhances TA and exhibits elevated expression in lung adenocarcinoma and breast cancer. Originating from the right arm of the terminal hairpin within TERC's H/ACA motif, TERC-sRNA, upon activation, facilitates recruitment of AGO2 and unidentified factor to TERC RNA [134]. This mechanism modulates AGO2's impact on TERC-TERT complex formation, thereby augmenting TA. Conversely, suppression of MYC activity leads to a decline in TERC levels, subsequently inhibiting tumor cell proliferation and suppressing tumor progression [129]. This phenomenon has similarly been observed in lung cancer research [133]. Upon knockout of the TERC gene, in vitro experiments demonstrate a reduction in tumor cell migratory capacity, accompanied by G1-phase cell cycle arrest [133]. Concurrently, animal studies illustrate suppressed growth of implanted tumors, further validating these findings [133]. Reduction in TERC expression not only diminishes its ability to complex with TERT for telomerase assembly but also impacts TERT's binding to genes such as c-Myc, cyclin D1, and VEGF, consequently attenuating the enzymatic activity and functional roles of telomerase [133]. In colorectal cancer research, Carriers of the common allele at SNP rs10936599, near the TERC locus, have significantly longer telomeres and a higher risk of colon cancer [135]. This suggests that the differences in TERC between individuals may be sufficient to affect the overall function of telomerase and even telomeres.

### Telomerase cofactors maintain the function of cancer cells

In cancer, the expression of telomerase accessory factors is generally elevated, and a reduction in their expression can lead to a range of functional impairments in cancer cells. The proteins NOP10, GAR1, NHP2, and dyskerin collectively form the dyskerin complex, which is indispensable for the proper folding and assembly of TERC [136–138]. The expression of these subunits is regulated at the transcriptional level by the upstream factor Ribosomal Protein S6 Kinase 2 (RIOK2). Deletions or mutations in the transcriptional domain of RIOK2, or the loss or mutation of any component of the dyskerin complex, may compromise the stability of TERC, leading to a reduction in telomerase activity and, ultimately, telomere shortening [139, 140]. Elevated levels of NOP10 have been linked to poor prognosis of breast and endometrial cancer [141, 142]. NOP10 serving as an independent prognostic marker, particularly in triple-negative breast cancer [141]. In studies focusing on NSCLC, it has been observed that elevated expression levels of the NOP10 protein are associated with enhanced cellular proliferation and migratory capacity. In non-small cell lung cancer (NSCLC), increased expression of NOP10 correlates with enhanced cellular proliferation and migratory capacity. Conversely, silencing NOP10 significantly reduces the expression of SNORA65, SNORA7A, and SNORA7B, as well as suppressing cancer cell proliferation and migration, ultimately decreasing TERC levels. These findings suggest that NOP10 plays a pivotal role in maintaining telomere activity in cancer cells, thereby facilitating tumor progression. Further investigations highlight that elevated gene expression in distinct cellular compartments may yield divergent effects on cancer cell behavior [143]. Research on GAR1 has also shown that its cytoplasmic positivity is detected in a subset of colorectal, liver, urothelial, and prostate cancers [144]. At the same time, low expression levels of these cofactors can be detrimental to human health. For instance, NHP2 is commonly overexpressed in colorectal cancer, particularly in elderly patients with colon cancer, and this overexpression correlates with a poor prognosis. When NHP2 expression is downregulated, tumor cells proliferation is inhibited. Deficiency in NHP2, which plays a critical role in ribosomal RNA biogenesis, can lead to serious conditions such as pulmonary fibrosis, a lung disease characterized by scarring of the lung tissue, and Høyeraal-Hreidarsson syndrome, a rare genetic disorder affecting multiple systems in the body [145]. Certain telomerase accessory factors exhibit variable expression across different cancers, with some factors showing increased expression, while others are downregulated. Dyskerin(DKC1), a core-component of the telomerase holoenzyme is typically overexpressed in breast and prostate cancers [146, 147], whereas its expression is often reduced in endometrial carcinoma and pituitary tumors [148, 149]. The downregulation of dyskerin impairs ribosomal protein synthesis and enhances the invasiveness of cancer cells [150]. Loss of dyskerin disrupts the translation initiation of tumor suppressors such as p53 and p27, potentially promoting tumorigenesis [151, 152]. Further research has shown that increased gene expression in different cellular compartments may have varying effects on cancer cells. TCAB1, also known as WRAP53 (WD repeat-containing, antisense to TP53), is one of the critical cofactors involved in telomerase function [153–155]. TCAB1 principally facilitates the recruitment of telomerase to the telomere. In the absence of TCAB1, telomerase, despite its overexpression, is incapable of performing its function at the telomere [156]. Overexpression of TCAB1 has been observed across various malignancies, including carcinoma, nasopharyngeal carcinoma, and other cancer types. Silencing TCAB1 in cancer cells has been shown to affect the ubiquitination and degradation of p21, leading to cell cycle arrest and the induction of cellular senescence in tumor cells [157]. Subcellular localization studies have demonstrated that the nuclear expression of TCAB1 confers a favorable prognosis in cancer patients [155]. Future research may focus on the differential subcellular localization of TCAB1 at various stages of disease progression, which could offer valuable insights into the underlying mechanisms of this paradox [158, 159]. Telomerase accessory factors may also exhibit mutations in cancer, leading to corresponding alterations. H2A and H2B form a dimer, referred to as the H2A-H2B dimer, which functions as an accessory factor for telomerase. This dimer may play a pivotal role in the folding of telomerase RNA and in enhancing the functional activity of the telomerase complex [160]. In uterine and ovarian sarcomas, an increase in mutations in the genes encoding H2A and H2B has been observed, and this mutation burden enhances the metastatic and invasive potential of the cancer cells [161].

### The non telomere oncogenic effects of telomerase

Telomerase and its associated components, beyond their canonical function in preserving telomere integrity, exert profound influence on cancer initiation and progression, with TERT assuming a particularly central role (Fig. 4B). TERT facilitates cancer cell proliferation via multiple pathways, including NF-κB, NRF2, MYC, P21, and NOP2 [162–167]. Notably, TERT upregulates the expression of NF-κB, NRF2, and MYC, which, in turn, reciprocally enhance TERT expression, forming a self-reinforcing positive feedback loop. Additionally, TERT augments VEGF expression and promotes angiogenesis through its interaction with SP1 or via the upregulation of HO-1 [168, 169]. ERT drives tumor cell invasion and metastasis through diverse mechanisms, including the upregulation of NF-κB, the induction and nuclear accumulation of β-catenin, and the increased expression of key transcription factors such as Snail1 and Snail2 [170–174]. In the realm of energy metabolism, TERT supports tumor growth by modulating glucose and fatty acid metabolic pathways. Furthermore, it facilitates immune evasion by enhancing the expression of CCL2, thereby recruiting myeloid-derived suppressor cells(MDSC) and regulatory T cells [175–177]. Lastly, TERT interacts with RPC32, a subunit of RNA polymerase III (pol III), to enhance pol III recruitment to chromatin. This interaction upregulates TERT-associated tRNAs in cancer cells, amplifying its pro-tumorigenic activities [178]. Wu et al. demonstrated that TERC overexpression induces the upregulation of the PDPK1 gene, subsequently activating AKT in a manner independent of telomerase activity [179]. Interestingly, AKT further augments TERC expression by inhibiting FOXO1 [179]. Moreover, TERC overexpression has been associated with increased EGFR mRNA levels [179]. Notably, previous studies have identified a correlation between the overexpression of ATK and EGFR poor prognostic outcomes in lung cancer and renal cell carcinoma [180–183]. These findings suggest that TERC may promote cancer initiation and progression through mechanisms independent of telomerase activity. The TERC primary transcript contains an alternative open reading frame that encodes a 13 kDa protein, known as human telomerase RNA protein, which confers protection against apoptosis [184]. While TERC knockdown does not significantly affect the viability of cancer cells, it sensitizes them to apoptosis induced by specific chemotherapeutic agents [185].

From a subcellular perspective, the functional roles of TERT vary depending on its intracellular localization. The subcellular distribution of TERT undergoes two distinct transitions: one involves its translocation from the cytoplasm to the nucleus, while the other entails its export from the nucleus to the cytoplasm. Typically, TERT is synthesized in the cytoplasm by ribosomes before translocating to the nucleus, where it exerts its functional effects [186]. The nuclear export factor NXF1 is essential for the translocation of both TERC and other long noncoding RNAs (lncRNAs) as well as messenger RNAs (mRNAs) from the nucleus to the cytoplasm. Upon its arrival in the cytoplasm, TERC associates with TERT and other components to form the telomerase complex, which subsequently reenters the nucleus [133]. The ability of TERT to maintain telomere length and regulate growth-related genes, which in turn promotes cancer cell proliferation, as well as its influence on downstream pathways that induce glycolysis, thereby facilitating cancer cell survival, metastasis, and invasion, are all intrinsically linked to TERT’s capacity for translocating in and out of the nucleus [187–189]. Studies have demonstrated that impairment of telomerase activity, such as through dephosphorylation by FBP1, which disrupts its shuttling between the nucleus and cytoplasm, renders cells more susceptible to genotoxic stress and can impede the achievement of cellular immortalization [190–193].

Typically, TERT and telomerase are localized within the nucleus, although they can occasionally be found in the cytoplasm [177]. At the subcellular level, upon exposure to exogenous stress, TERT transfer from the nucleus to mitochondria in cancer cells [194]. At the subcellular level, upon exposure to exogenous stress, TERT transfer from the nucleus to mitochondria in cancer cells [195–199]. Furthermore, telomerase within mitochondria protects mitochondrial function and inhibits apoptosis [190, 191]. This suggests that the subcellular distribution of telomerase and TERT is intricately linked to the survival of cancer cells. For example, when exposed to oxidative stress such as H_2_O_2_, cancer cells that release telomerase from their nuclei will show minimal or no DNA damage [200]. The process of telomerase translocation may have three significant protective roles for the cell: first, it can protect mitochondrial function; second, it can reduce the production of mitochondrial superoxides, significantly lowering ROS levels and protecting the nucleus from DNA damage; third, it may prevent adverse effects of telomerase on repair enzymes within the nucleus, thereby avoiding erroneous chromosomal repair [200]. The specific mechanisms involve TERT binding to the ND1 and ND2 coding regions of mtDNA within the mitochondrial matrix under conditions of oxidative stress, thereby protecting mtDNA from oxidative damage and maintaining genomic integrity [48, 198]. Additionally, TERT upregulates superoxide dismutase and components of the electron transport chain to mitigate the production of ROS [199, 201]. TERT also interacts with coactivator 1 α and β to regulate the transcription of mtDNA, which is a critical determinant of cellular metabolism and is closely associated with aging and cancer [48, 202]. The extrusion of TERT from the nucleus may be related to the presence of the tyrosine phosphatase Shp-2, whose function is to retain TERT within the nucleus [202]. Under conditions of oxidative stress, a reduction in Shp-2 expression may lead to the translocation of nuclear TERT out of the nucleus [203]. This phenomenon may contribute to the observed chemoresistance in studies of cancers such as HCC and breast cancer [189, 204].

However, some studies have indicated that, in certain cancer cell types, TERT is predominantly localized to the cytoplasm. This subcellular localization of TERT may also play a critical role in promoting metastasis. In human nasopharyngeal carcinoma (NPC), for instance, TERT is mainly found in the cytoplasm of tumor cells, while in lymph node metastases, it is predominantly localized in the nucleus [205]. This shift from cytoplasmic to nuclear localization may be induced by the phosphorylation of Akt and protein kinase C [205, 206]. The mechanism by which TERT facilitates metastasis likely involves its regulation of genes encoding proteins such as TGF-β and β-catenin, both of which are implicated in NPC invasion and metastasis [205, 207]. Notably, the nuclear accumulation and expression of hTERT in cancer cells can enhance the nuclear translocation of β-catenin [164, 177]. In HCT116 human colon cancer cells, overexpression and nuclear accumulation of hTERT promote β-catenin’s nuclear translocation and its increased expression, leading to co-expression with TCF-4. This interaction forms the β-catenin/TCF-4 complex, which subsequently activates the expression of the Chemokine (C–C motif) ligand 2 (CCL2) promoter [208–211]. Elevated serum levels of CCL2 can recruit myeloid-derived suppressor cells (MDSCs), thereby promoting tumor cell proliferation and metastasis. Moreover, high serum concentrations of CCL2 have been associated with metastasis in various cancers, including colorectal and lung cancer, and correlate with poorer patient prognosis [212, 213].

## Telomeres and telomerase as cancer biomarkers

### Telomere as cancer biomarkers

Given the critical role of telomeres and telomerase in carcinogenesis, utilizing them as biomarkers may enhance our ability to predict the onset and progression of cancer, as well as prognosis, thereby aiding clinicians in devising optimal treatment strategies.

The main indicators include telomere length (TL) and telomere length variation (TLV) (Table 1). In early studies, researchers often used telomeres or telomerase from a single cell type as biomarkers. Ma LJ et al. demonstrated that shorter telomeres in liver cancer patients or in cancer-associated fibroblasts (CAFs) are often indicative of a higher risk of mortality and recurrence [214]. Hamada T et al. showed that shorter leukocyte telomeres prior to diagnosis in pancreatic cancer patients were associated with a reduction in survival time of approximately three months [215]. This may be attributable to chromosomal instability and DNA damage induced by shortened telomeres, leading to gene mutations that contribute to tumor malignancy [216, 217]. When assessing telomere length, researchers typically rank telomeres from shortest to longest and then divide them into quartiles to determine which telomeres are relatively short and which are relatively long [218, 219]. Describing data characteristics through quartiles is a commonly employed method in medical research [220]. Short leukocyte telomere length (LTL) can be associated with cancers such as colorectal cancer, ovarian cancer. Some cancer studies have shown that longer leukocyte or relevant organization TL increase the risk of death from various cancers, such as prostate cancer [158, 221]. Serum cell-free DNA (cfDNA) may be released from necrotic, apoptotic, and tumor cells during inflammatory and carcinogenic processes [222]. Research has demonstrated that assessing telomere length through cfDNA in gastric cancer reveals a significant correlation between telomere shortening and an elevated risk of disease progression [223]. There are also studies suggesting that LTL is related to the risk of cancer, such as lung cancer, breast cancer [218, 219]. Longer LTL increase the incidence rate of cancer and the risk of death, while reducing the probability of other diseases and their associated mortality [224]. Sometimes, the relationship between telomere length and pancreatic cancer risk is U-shaped. Skinner HG et al. showed that when subjects were ranked from shortest to longest LTL, those in the 1% to 90% percentile displayed an increased risk of cancer with shorter telomeres [225]. However, individuals in the top 1% with the longest telomeres had a higher risk of pancreatic cancer. There is also gastric cancer with a similar structure [226]. This may explain why, within the same type of cancer, some studies suggest that shorter telomeres are associated with a higher risk of death, whereas others find that longer telomeres are linked to an increased risk of mortality. However, these studies seem to have not included detection of P53 activity, which may determine the relationship between LTL length and cancer.

**Table 1:** Assays utilizing telomeres as a biomarker

Types of cancer	Total sample size	Types of biomarkers	Finding	Research year	Reference
Breast cancer	440	TL	Telomere shortening of 9p, 15p, 15q and Xp in blood lymphocytes is associated with the onset of breast cancer in pre-menopausal women	2010	[229]
Pancreatic cancer	1462	TL	Short LTL and extremely long LTL have the highest risk of pancreatic cancer	2012	[225]
Lung cancer	398	TL, TLV	The length of telomeres in blood cells varies greatly, and shorter telomeres are a risk factor for patients under 60 years old and a protective factor for patients over 60 years old	2015	[228]
Gastric cancer	2148	TL	Both short and extremely long LTL are risk factors for gastric cancer	2015	[226]
Liver cancer	257	TL	A shorter TL for HCC or CAFs indicates a higher risk of recurrence and mortality	2017	[214]
Ovarian cancer	1169	TL	Shortet LTL increase the risk of ovarian cancer	2018	[232]
Prostate cancer	535	TL	Longer LTL increases the risk of prostate cancer death	2018	[158]
Breast cancer	442	TL	Longer LTL is associated with breast cancer risk	2019	[218]
Gastric cancer	159	TL	Shorter cfDNA TL is associated with the progression of gastric cancer	2024	[223]
Pancreatic cancer	423	TL	Shorter LTL at diagnosis are associated with shorter survival times	2020	[215]
Mixed study of multiple types of cancer	77	TL	Longer LTL increases the risk of cancer incidence rate and death	2021	[224]
Prostate cancer	1048	TL	Longer prostate TL is associated with prostate cancer risk	2022	[221]
Prostate cancer	2255	TV, TLV	The combination of more variable telomere length among prostate cancer cells and shorter telomere length in prostate cancer-associated stromal cells is strongly associated with progression to metastasis and prostate cancer death	2022	[227]
Colorectal	198	TL	Shorter LTL increase the risk of colorectal cancer	2023	[233]
Lung cancer	1913	TL	Long LTL in non-smoking individuals increases the risk of lung cancer	2023	[219]
Mixed study of multiple types of cancer	1434	TL	The longer the telomeres of cancer cells and cancer-associated fibroblasts, the higher the degree of cancer deterioration and the worse the prognosis	2023	[234]

*TL* telomere length* LTL* Leukocyte telomere length, *TLV* Telomere length variation

Subsequently, it was recognized that a single biomarker cannot fully explain subsequent developments, leading to the exploration of combining multiple biomarkers from the same cell type or different cell types as a potential direction for cancer biomarker research. In prostate cancer research, Hifi CM found that the combination of more variable telomere length in prostate cancer cells and shorter telomere length in prostate cancer-associated stromal cells is strongly associated with progression to metastasis and cancer mortality [227]. Due to the close relationship between telomeres and aging, age may also influence the applicability of telomeres as biomarker. In the study by Sun B et al., it was shown that greater variability in telomere length and shorter telomeres in blood lymphocytes are positively associated with lung cancer risk in patients under 60 years old, but negatively correlated with lung cancer risk in patients over 60 years old [228]. However, the significance of telomere length may vary across different chromosomes. Zheng YL et al. studied telomeres in blood lymphocytes and found that only shortened telomeres on 9p, 15p, 15q, and Xp were associated with an increased risk of breast cancer in pre-menopausal women [229]. In terms of telomere length, the significance of telomere elongation or shortening may vary among different cancers due to the diverse cell types involved. Additionally, telomere length is influenced by genetic variation, environmental factors, and therapeutic interventions, among others. Some factors have differing short-term and long-term effects on telomeres dynamics. For example, Hou et al. showed that long-term exposure to air pollution may shorten blood telomeres, whereas short-term exposure might actually lengthen them [230]. Research by Shoeb et al. has also indicated that exposure to welding fumes may alter the expression of TPP1, POT1, and RAP1 within the shelterin complex, leading to telomere elongation in the presence of normal TERT levels, which can increase the risk of lung carcinogenesis [231]. Therefore, these factors should be considered when using telomeres as biomarkers. It may be advisable to combine multiple biomarkers for predictive analysis.

### Telomerase as cancer biomarkers

Telomerase, as a biomarker, is often detected indirectly through methods such as detecting telomere repeat sequences through TRAP or detecting the expression level of TERT (Table 2). When comparing breast tissue from breast cancer patients to healthy individuals, Kulić A et al. demonstrated that the median TA in the patients' breast tissue was nearly 20 times higher (1.032 vs. 0.053) compared to normal breast tissue [235]. This study also demonstrated that TA levels in cancer patients were significantly and positively correlated with tumor size, histological grade, axillary lymph node status, nuclear antigen Ki-67, and HER-2/neu protein expression. This may explain why patients with higher TA levels have a shorter 10-year disease-free survival. In gliomas, the overall survival (OS) (10.9 vs. 15.9) and progression-free survival (PFS) (6.9 vs. 12.3) of the TERT promoter mutant group were significantly lower than those of the TERT promoter wild-type group, representing a poorer prognosis [236]. There is consistent evidence for predicting patient survival rates through tumor tissue in other cancers such as thyroid cancer and lung cancer. The level of TERT expression, which represents TA, is a more suitable biomarker for disease-specific survival in patients with urothelial cancer than promoter mutations [237]. However, TERT promoter mutations may still serve as a biomarker, with 26.7% of sebaceous carcinomas showing promoter mutations detected, whereas no such mutations were found in sebaceous adenomas [238]. From the perspective of TERT, both THOR and the C288T mutation site can serve as biomarkers. Apolónio JD et al.'s study showed that the methylation probability of THOR in malignant breast tissue was 40%, while in benign tissue it was 12.81%. The C228T mutation in TERT is present in 76.47% of primary glioblastoma patients and is associated with poor prognosis. Therefore, this mutation can serve as a key follow-up biomarker and a novel therapeutic target [239]. Another common mutation site in TERT also shows potential as a biomarker in chondrosarcoma. Zhang Y et al. demonstrated that the C124T mutation is present in 45% of chondrosarcoma patients and is associated with signs of transformation, early metastasis, or late but aggressive metastatic patterns [240]. From the above studies, it can be observed that whenever a mutation is used as a biomarker, it generally correlates with poorer outcomes. However, Thielmann CM's research shows that TERT promoter mutations are associated with longer asymptomatic survival in BRAF mutant melanoma patients treated with BRAF and MEK inhibitors [241]. Kim SK's study found that TERT promoter mutations are associated with low histological grade, low mitotic count, absence of necrosis, low Ki-67/MIB-1 labeling index, and lack of lymph node or distant metastasis. These findings may require validation in larger-scale studies with more participants, but they suggest that the role of the TERT promoter in cancer warrants further investigation [242].

**Table 2:** Assays utilizing telomerase as a biomarker

Types of cancer	Total sample size	Types of biomarkers	Finding	Research year	Reference
Head and Neck Cancer	120	TA	Telomerase positivity in PBMCs is associated with head and neck cancer	2006	[246]
Urothelial cancer	122	TAL	Elevated TERT expression correlates with decreased DSS	2015	[237]
Breast cancer	122	TAL	Higher TAL are associated with more severe breast cancer and shorter survival periods	2016	[235]
Gastrointestinal Cancer	131	TERT promoter methylation	TERT promoter methylation in feces is associated with GIC	2017	[247]
Mixed study of multiple types of cancer	178	TERT mRNA	Detection of TERT mRNA positivity in exosomes within serum indicates the presence of cancer in patients	2017	[253]
HCC	316	TERT's positioning	High cytoplasmic TERT in NCHCC and low cytoplasmic TERT in CCHCC indicate better prognosis	2017	[243]
Gliomas	71	TERT promoter mutations	TERT promoter mutations are associated with shorter OS and PFS	2018	[236]
Ovarian cancer	49	TERT promoter methylation	The frequency of TERT promoter methylation in ctDNA derived from tumor tissue is significantly higher than that observed in benign tissue	2020	[250]
Glioblastoma	85	TERT promoter mutations	The C228T mutation in TERT can be used for the diagnosis of primary glioblastoma patients and is associated with poor prognosis	2021	[239]
Sebaceous carcinomas	91	TERT promoter mutations	TERT promoter mutation can help distinguish between sebaceous gland tumors and sebaceous gland cancers	2021	[238]
Chondrosarcoma	190	TERT promoter mutations	The C124T mutation in TERT can be used for the diagnosis of primary Chondrosarcoma patients and is associated with poor prognosis	2021	[240]
Penile squamous cell carcinoma	57	TERT promoter mutations	TERT promoter mutations are well-correlated with favorable clinical and pathological grading."	2021	[242]
Melanoma	232	TERT promoter mutations	TERT promoter mutations are associated with improved survival in BRAF-mutated melanoma patients treated with BRAF and MEK inhibitors	2021	[241]
Bladder cancer	113	TA	Telomerase positivity is associated with bladder cancer, and TA can help in grading the malignancy of tumors	2021	[251]
Breast cancer	17	THOR methylation	THOR methylation can help distinguish malignant breast tissue from benign tissue	2022	[257]
Breast cancer	144	TERT's positioning	High cytoplasmic TERT in HER2-positive breast cancer cells correlates with drug resistance	2023	[245]
Brain tumors	276	TERT mRNA	Elevated levels of TERT mRNA in serum exosomes correlate with higher grades of brain tumors	2024	[254]
Breast cancer	15	TERT promoter mutations	The loss of cfDNA promoter methylation indicates an effective therapeutic response	2024	[248]
HCC	502	TERT promoter mutations	A higher frequency of promoter mutations in ctDNA is associated with advanced staging and increased mortality in hepatocellular carcinoma	2024	[250]

*TAL* telomerase activity levels,* PBMC*peripheral blood mononuclear cells,* DSS*disease-s pecific survival,* NCHCC*hepatocellular carcinoma* CCHCC* clear cell hepatocellular carcinoma, *THOR*, *TERT* hypermethylated oncological region,* GIC* gastrointestinal cancer* HER2* human epidermal growth factor receptor 2.* HCC* hepatocellular carcinom

From a subcellular perspective, the localization of TERT within cancer cells can serve as a biomarker. Huang et al. conducted a study involving 259 patients with non-clear cell hepatocellular carcinoma (NCHCC) and 57 patients with clear cell hepatocellular carcinoma (CCHCC) [243]. Their findings indicated that among NCHCC patients, those exhibiting low nuclear and high cytoplasmic expression of TERT tended to have better recurrence-free survival [243]. Conversely, individuals with high nuclear expression had the poorest prognosis [243]. In CCHCC patients, low expression of TERT in both the nucleus and cytoplasm was associated with a favorable prognosis, whereas high cytoplasmic expression correlated with the worst recurrence-free survival [243]. However, the study by Nishi et al. did not find an association between the subcellular localization of TERT and the prognosis of patients with HCC; this discrepancy may be related to the specific subtype of cancer in the patient population [244]. Uno et al. conducted a subcellular localization analysis of TERT in 114 patients with breast cancer, specifically examining the luminal A (LA), luminal B (LB), HER2-overexpressing (HER2), and triple-negative (TN) subtypes [245]. They only reported that, in the HER2 subtype, there was a correlation between TERT localization and breast cancer. Prior to primary systemic therapy (PST), the immunohistochemical nuclear expression of TERT was significantly higher in the HER2 subtype compared with the other subtypes, whereas there was no notable difference in cytoplasmic TERT expression [245]. Notably, 41.7% of patients with the HER2 subtype exhibited TERT expression exclusively in the nucleus. Following PST, many cases showed increased cytoplasmic TERT expression in breast cancer cells, which was associated with resistance [245]. This suggests that when considering TERT subcellular localization as a biomarker, the specific subtype of cancer must be taken into account.

The detection of biomarkers related to telomerase can be done not only through tissues, but also through feces and urine. Lee et al. demonstrated that in head and neck cancer studies, the expression of telomerase in peripheral blood mononuclear cells (PBMCs) can serve as a biomarker for diagnosis. Among 100 patients, 73 (73.0%) were telomerase-positive, showing a statistically significant difference compared to healthy individuals [246]. Liu et al. detected TERT promoter methylation in the feces of patients with gastrointestinal Cancer (GIC) and healthy individuals, finding that the results were useful for GIC screening [247]. In breast cancer, a reduction in TERT methylation levels in cfDNA has been observed following effective treatment, suggesting that cfDNA analysis could serve as a potential biomarker for monitoring therapeutic efficacy in the future [248]. After isolating the DNA fragments derived from tumor cells within cfDNA, they are categorized as circulating tumor DNA (ctDNA). Through the analysis of ctDNA, mutations and methylation in the TERT promoter can be detected, serving as valuable biomarkers for assessing disease progression [249, 250]. Glukhov et al. demonstrated that TA in urine can aid in diagnosing bladder cancer, although this detection method has limitations. Specifically, some cancer patients show negative TA in their urine, and some non-cancerous patients with inflammation may present positive results [251]. According to the research conducted by Gutkin et al., telomerase within exosomes derived from cancer cells retains its enzymatic activity, which can act on other cells, such as fibroblasts, extending their lifespan. hTERT mRNA was detected in these exosomes from cancer cells [252]. The study by Goldvaser et al. was the first to demonstrate hTERT positivity in serum exosomes among patients with various types of cancer. Although not all patients exhibited this positivity, all healthy individuals tested negative, suggesting that this may serve as a novel biomarker for cancer diagnosis [253]. The study by Uziel et al. demonstrated that the levels of TERT mRNA in serum exosomes correlate with the malignancy grade of primary brain tumors [254]. Rotem et al. also validated the potential of TERT mRNA as a biomarker in extracellular vesicles in the context of lung cancer [255]. The study by Laish et al. suggests that the levels of exosomal serum hTERT mRNA are associated with metastatic colorectal cancer [256].

### Heterogeneity of telomeres and telomerase in cancer

#### Breast cancer

In breast cancer cells, TA is nearly ubiquitous across all subtypes of the disease, with a prevalence approaching 100% [258, 259]. Therefore, telomerase is an important target for different subtypes of breast cancer [260]. However, studies have reported that the proportion of breast cancer patients exhibiting TA ranges from approximately 50% to 80% [261, 262]. The expression of TERT in breast cancer cells may be subject to direct or indirect modulation by various signaling pathways, including estrogen receptor (ER), progesterone receptor (PR), and human epidermal growth factor receptor 2 (HER2), leading to differential manifestations of disease within the same subtype of breast cancer. The positive expression of hTERT exhibits a statistically significant correlation with HER2, yet no significant association with estrogen receptor (ER) or progesterone receptor (PR), nor with tumor size or histological grade [261, 263]. This phenomenon occurs because overexpression of HER2, similar to RAS and RAF, can induce hTERT mRNA via the ERK MAP kinase pathway and the ETS transcription factor ER81, thereby activating telomerase in cells from a negative to a positive state [264]. In breast cancer, TERT promoter mutations are exceptionally rare, occurring in only 0.9% of tumor tissues. Notably, within the rare phyllodes tumor subtype, more than 50% exhibit the promoter − 124 C > T hotspot mutation and/or TERT gene amplification. This leads to increased TERT expression, which is correlated with histologic grading [265, 266]. The relationship between HER2 overexpression and telomere shortening warrants further investigation, given the conflicting results across different studies. Helal et al. demonstrated an association between HER2 overexpression and telomere length in peripheral blood leukocytes prior to treatment, whereas Murillo-Ortiz et al. did not find such an association and instead noted correlations with telomere length post-treatment [261, 267]. Furthermore, Helal et al. observed that shorter telomeres were associated with shorter OS, higher malignancy, and greater lymph node involvement [267]. Meeker al. showed that telomeres in Luminal A cases are generally longer than those in Luminal B, HER2-positive, and triple-negative cases [268]. The investigation conducted by Heaphy et al. further revealed that within a cohort of neoplastic tissues characterized by shortened telomeres, there was a heightened prevalence of ER and PR negativity. Additionally, an elevated incidence of p53 positivity was noted, contributing to genomic instability [269]. However, Lu et al. indicated that telomere length in tumor tissues is not correlated with clinical outcomes or characteristics in breast cancer [270]. The relationship between these findings and factors such as ethnicity and exposure requires further investigation to be conclusively established. Intriguingly, these molecular distinctions may serve to elucidate the clinical characteristics associated with histological variations. From a histological classification perspective, the proportion of short telomeres in invasive lobular carcinoma (ILC) is approximately 48%, significantly lower than the 85% observed in invasive ductal carcinoma (IDC) [271]. When considering the molecular subtypes of triple-negative breast cancer (TNBC), the frequency of short telomeres in ILC is 40%, markedly lower than the 95% noted in LDC [271]. Furthermore, only 2% of ILC cases exhibit telomeres that are notably shorter than those in other ILC cases, a phenomenon that may be attributed to the fact that approximately 90% of ILC cases are ER + [271].

#### Lung cancer

Through statistical analysis of an untreated population, it was found that individuals with longer leukocyte telomeres may be at a higher risk for lung cancer, particularly among nonsmokers [272]. Since smoking, a significant etiological factor for lung cancer, also leads to telomere shortening, it likely masks the increased risk associated with longer telomeres. This study circumvents the confounding effect of telomere shortening due to cancer therapy [272]. From a histopathological perspective, individuals with longer telomeres appear to have a particularly strong association with an increased risk of adenocarcinoma, a subtype of lung cancer [272, 273]. This phenomenon may be attributable to the fact that human telomeres are optimally maintained at a certain length; telomeres that are excessively long may contribute to chromosomal instability during their shortening, leading to the accumulation of mutations and the development of cancer [274, 275]. Studies have shown that telomerase expression in NSCLC tissues is approximately 70%, whereas it is absent in normal lung tissue. Similarly, 70% of NSCLC tissues exhibit hTERT positivity, compared with only about 15% in normal lung tissue [276, 277]. These markers are useful in distinguishing neoplastic from non-neoplastic tissues. However, the significance of these indicators in relation to tumor staging, differentiation, lymph node metastasis, and prognosis remains a subject of debate [278]. Studies by Hiyama et al. have shown that, regardless of whether the neoplasm is an in situ carcinoma or a metastatic lesion, approximately 20% of lung cancer specimens lack measurable TA. In contrast, small cell lung cancers (SCLC) invariably display robust TA [279]. In 98% of human SCLC, TERC is upregulated [280]. In contrast, TERC expression in NSCLC is considerably lower. Notably, the positivity rate for hTERC in squamous cell carcinoma is substantially higher than that in other subtypes of NSCLC. Specifically, the hTERC positivity rate in squamous cell carcinoma is 41%, whereas the combined positivity rate for adenocarcinoma and large cell carcinoma is only 8% [281]. Pulmonary sarcomatoid carcinoma (PSC) is a highly disseminated and relatively uncommon subtype of NSCLC [282]. Despite employing treatment strategies similar to those used for NSCLC, outcomes remain suboptimal, and the prognosis for PSC is exceedingly poor [282]. This disease is characterized by mutations in POT1, which are not typically encountered in other forms of lung cancer [102]. Approximately 83% of these mutations affect the OB1 and OB2 domains of POT1, potentially leading to aberrant activation of ATR. When coupled with defects in p53, such activation may accelerate the progression of the disease [102, 283]. The remaining mutations are found in the OB3 domain. These findings suggest that ATR kinase inhibitors may offer promising therapeutic potential in the treatment of this particular lung cancer subtype [102].

#### Leukemia

Given the presence of TA in hematopoietic stem cells, targeted telomerase therapies for leukemia may require greater consideration compared with those for other cancers [284]. These inhibitors may also lead to telomere shortening, senescence, and apoptosis in normal cells [285]. In acute leukemia (AL), both acute myeloid leukemia (AML) and acute lymphoblastic leukemia (ALL) exhibit elevated telomerase activity in peripheral blood mononuclear cells or hematopoietic progenitor cells, levels that are markedly greater than those observed in healthy individuals—often more than tenfold and, in some cases, up to 50-fold [286]. However, this is not universally observed; among all patients with acute leukemia, 75 percent exhibit elevated telomerase activity, marked upregulation of TERT expression, and a general shortening of telomere length [286–291]. In ALL expressing BCR-ABL1, this subtype may activate the PI3K/Akt/mTOR signaling pathway, leading to the induction of hTERT transcription by NFAT and c-Myc. Additionally, this process may involve partial induction of nuclear translocation via NF-κB, thereby enhancing telomerase activity and ultimately resulting in a poor prognosis [292–295]. Furthermore, patients with recurrent disease exhibit the highest levels of TA, followed by those at initial diagnosis, with the lowest levels observed in the treated group [286]. This suggests that TA may serve as a marker for disease recurrence. TA is noted to increase during the G1, S, G2, and M phases of the cell cycle, whereas it diminishes in the G0 phase [286]. Acute promyelocytic leukemia (APL), a subtype of acute myeloid leukemia (AML), exhibits superior clinical outcomes and prognosis compared with other forms of AML. This may be attributed to the fact that TA in APL is only one-quarter that of other AML subtypes [286]. Patients with higher TA exhibit significantly lower 5-year OS rates compared with those who have lower TA levels. Therefore, telomerase activity may serve as a prognostic indicator in AL [286]. Regarding hTERT expression levels, the trend is B-Acute Lymphoblastic Leukemia (B-ALL) > T-Acute Lymphoblastic Leukemia (T-ALL) > AML [296]. Differences in TA may be attributable to the presence of distinct hTERT transcript isoforms among different subtypes of leukemia. For instance, normal bone marrow mononuclear cells (BMMNC) exhibit a higher expression of the –α + β isoform, whereas B-ALL cells show a higher expression of the –α-β isoform. These isoforms exert regulatory effects on TA, with the former possessing an inhibitory action and the latter being inactive [296, 297]. In B-ALL, the upregulation of TA may be associated with β-Arrestin1 facilitating the interaction between P300 and Sp1, a process that can enhance the binding of Sp1 to the hTERT promoter, thereby increasing hTERT transcription [292]. This observation suggests that hTERT may undergo post-transcriptional modifications [296]. In T-ALL, the onset of the disease may not necessitate telomerase activity; rather, the activation of telomerase appears to play a role primarily in the maintenance and progression of the disease [298]. High expression of TERT is not closely associated with TERT promoter mutations, since such mutations are more frequently observed in solid tumors, whereas reports of these mutations in hematologic cancers are rare [299]. Furthermore, TL shows a significant negative correlation with TA. Consequently, TL may also serve as an indicator for disease recurrence or prognosis [286]. In acute leukemia, there are variations in telomere length among different subtypes, with the overall trend being AML > T-ALL > B-ALL [296]. Moreover, in AML and ALL, abnormal leukemic cells exhibit shorter telomeres compared with those possessing a normal karyotype [296, 300]. Within these subgroups, there are expression differences in the telomeric genes TPP1, RAP1, and TRF2. Expression levels of TPP1 and RAP1 in T-ALL are approximately twice those observed in B-ALL and AML. In contrast, B-ALL exhibits TRF2 expression levels that are more than 8 times higher than those in T-ALL and AML. This suggests that TRF2 may be an effective therapeutic target in B-ALL [296]. In patients with chronic leukemia(CL), telomerase activity is elevated only 2 to 5 times above that of normal cells [290]. Similarly, chronic lymphocytic leukemia (CLL) shares features with ALL, such as high TA and shorter TL, which may contribute to a poorer prognosis [301, 302]. In chronic myeloid leukemia (CML), TA progressively increases through the chronic phase (CP), accelerated phase (AP), and blast crisis phase (BP) [303]. In CLL, activation of the TERT promoter is predominantly mediated through the JAK2/STAT3 signaling pathway, and TA is regulated via the HIF-1α pathway [304, 305]. Furthermore, POT1 mutations are present in only 3.5% of CLL cases, a finding not observed in other subtypes. This mutation is indicative of poor prognosis and is associated with a greater number of chromosomal abnormalities [306]. An increased TL is associated with an elevated risk of acute leukemia (AL) and CLL, whereas no significant correlation is observed with CML.

#### Glioma tumor

Mutations in RAS, TERT, and p53 synergistically drive glioma formation [307]. Aberrant activation of RAS promotes cellular proliferation and survival through the PI3K/AKT and MAPK/ERK signaling pathways [308, 309]. Concurrently, mutations in the TERT promoter sustain telomere length, thereby endowing cells with the capacity for limitless replication. The loss of p53 impairs apoptotic pathways and DNA damage repair mechanisms, further facilitating tumor progression. Additionally, p53 interacts with the transcription factor NF-Y, enhancing the ZDHHC5-mediated palmitoylation of EZH2 [307] Palmitoylated EZH2 inhibits the binding of DNMT3A to the OCT4 promoter, thereby reducing its methylation and promoting the overexpression of OCT4, which in turn accelerates glioma progression [307]. The relationship between TL and the risk of glioma is not linear; the highest risk is observed in the tertile with the longest telomeres, followed by the tertile with the shortest telomeres [310]. Alleles in the TERC and TERT regions are consistently associated with an increased risk of glioma and longer LTL, such as those at rs1920116 and rs2736100. In contrast, certain glioma risk alleles near the RTEL1 locus are predominantly linked with shorter LTL, as exemplified by rs6010620 [311]. The frequency of TERT promoter mutations varies among different grades of gliomas, occurring in approximately 45% of grade 2 and grade 3 gliomas and in about 75% of grade 4 glioblastomas [312]. In primary glioblastoma and oligodendroglioma, the frequency of TERT promoter mutations is approximately 80%, whereas in astrocytoma, the incidence of such mutations is only about 10% [117]. Furthermore, more than 70% of grade 2 and grade 3 gliomas harbor mutations in the IDH gene, suggesting a more complex relationship between glioma risk polymorphisms and mechanisms of telomere maintenance that requires further investigation [312, 313]. However, stratification based on the combined molecular biomarkers TERT and IDH can provide valuable insights for predicting patient survival outcomes. Among glioma patients, 26.47% belong to the IDHmut-TERTmut subgroup, which is associated with the most favorable overall survival. In contrast, the IDHwt-TERTmut subgroup, representing 20.17% of cases, is linked to the poorest survival outcomes [314]. Patients with higher TA tend to have a poorer prognosis [315].

#### Melanoma

Melanoma in its non-invasive form is classified as melanoma in situ (MIS). More than half of MIS patients have promoter mutations [316]. However, once it acquires invasive potential, it is subdivided into five major histological subtypes. Among these, superficial spreading melanoma (SSM) constitutes 41%, nodular melanoma (NM) accounts for 16%, lentigo maligna melanoma (LMM) represents 2.7% to 14%, uveal melanoma (UM) comprises 3% to 5%, and acral lentiginous melanoma (ALM) occurs in 1% to 5% of cases [317–321]. In melanoma, the aberrant overexpression of TERT is predominantly driven by three mechanisms: promoter mutations, TERT amplification, and enhancer hijacking [322]. From the perspective of the lesion site, the majority of TERT promoter mutations occur at positions -124 and -146, with these mutations being most prevalent in cutaneous melanoma (CM), accounting for 84% of cases, compared to 15% in mucosal melanoma (MM) and 10% in acral melanoma (AM). In contrast, enhancer hijacking and TERT amplification are more commonly observed in AM, with enhancer hijacking present in 29% of AM, 13% of MM, and 4% of CM cases. TERT amplification is found in 17% of AM, 12% of MM, and only 2% of CM cases [322]. The TERT-124 [C > T] mutation is predominantly observed in non-cutaneous cancers, whereas the TERT-146 [C > T] mutation is more commonly associated with cutaneous cancers. Melanomas arising in patients with TERT promoter mutations are frequently associated with increased Breslow thickness and a correspondingly worse prognosis [323]. In particular, the TERT-124 [C > T] promoter mutation may be linked to heightened aggressiveness, contributing to the tumor's more invasive characteristics [324]. The variant associated with the poorest prognosis is the 138/-139 CC > TT mutation, accounting for approximately 5% of cases [325]. C > T mutations, particularly the CC > TT variant, are strongly associated with ultraviolet (UV) exposure [326]. This partly explains why CC > TT changes are less likely to originate from internal organs and more likely to originate from the skin [326]. The longer an individual's telomeres, the higher their risk of developing melanoma. In terms of telomere length, individuals with longer telomeres prior to malignant transformation exhibit a higher risk of developing melanoma [327]. Following malignant transformation, TL from shortest to longest is CM, AM, MM, and UM [322]. In melanoma, promoter mutations alone may be insufficient to extend the telomeres in all types of melanoma cells. In some instances, additional genetic abnormalities are required to act in concert, influencing telomere dynamics and enabling cellular immortality [328]. A variant within the ACD region, which leads to increased expression of the TPP1 gene, has been identified as a collaborating mutation [94]. This variant occurs in approximately 5% of melanoma cases and helps explain why, during the early transitional stages of melanoma, telomeres in cells with TERT promoter mutations may continue to shorten, despite the mutation’s presence.

#### Gastric cancer

In gastric cancer, shorter telomere length is associated with an increased risk of disease, potentially due to the high turnover rate of epithelial cells in the stomach, one of the most frequently regenerating tissues [329, 330]. Studies have also indicated that excessively long telomeres are associated with an increased risk of gastric cancer [331]. Although TERT promoter methylation occurs in normal gastric mucosal samples, it does not result in TERT expression [332]. In gastric cancer samples, not only is the frequency of TERT promoter methylation significantly higher than in normal gastric mucosa, but 80% of these cancer samples also exhibit TERT expression [332]. In normal gastric mucosal cells, the transcription factor Early B-cell factor 1 (EBF1) suppresses TERT expression [333]. However, in gastric cancer cells, multiple mechanisms inhibit the normal function of EBF1 [333]. Firstly, epigenetic silencing of EBF1 expression is mediated by Polycomb Repressive Complex 2 (PRC2), DNA methyltransferases, and histone deacetylases (HDACs). Secondly, deletions or rearrangements occur at the EBF1 binding site proximal to the TERT promoter in gastric cancer cells. Lastly, mutations in EBF1 are observed in some gastric cancer cells. Together, these three mechanisms lead to the reactivation of TERT [333]. The non-telomeric functions of hTERT promote oncogenesis by forming a complex with the oncogene MDM2 [334]. This complex enhances the ubiquitination of FOXO3a, a member of the forkhead box O family, leading to its degradation [334]. Consequently, the inhibitory effect of FOXO3a on integrin β1 (ITGB1) expression is reduced, resulting in elevated ITGB1 levels and increased invasiveness of gastric cancer cells [334]. FOXO3a is involved in regulating tumor energy metabolism and development, while ITGB1 plays a role in cell adhesion, proliferation, and differentiation [335, 336]. In gastric cancer patients, hTERT expression positively correlates with ITGB1 expression, and both are associated with poor prognosis. Based on the Lauren classification, gastric cancer can be divided into three categories: intestinal-type gastric cancer, diffuse-type gastric cancer, and mixed-type gastric cancer. In intestinal-type gastric cancer, polymorphism in the second intron of chromosome 5p15.33, specifically rs2736100, can be detected, presenting in three genotypes: C/A, A/A, and C/C. The C/C genotype is associated with increased telomere length and elevated TERT mRNA levels [337]. In contrast, this polymorphism is not detectable in diffuse-type gastric cancer, which is also characterized by shorter telomeres [337]. Additionally, in intestinal-type gastric cancer, a positive correlation has been observed between telomere length and mitochondrial DNA (mtDNA) copy number, whereas no such correlation has been identified in diffuse-type gastric cancer [338]. Multilayered proteomic analysis suggests distinct pathogenic mechanisms between the two conditions: DNA damage is upregulated in intestinal-type gastric cancer, whereas immune and extracellular matrix (ECM) proteins are elevated in diffuse-type gastritis [339]. The link between DNA damage and telomere dynamics in intestinal-type gastric cancer may explain these observations.

#### Ovarian cancer

In most cancers, cancer cells utilize methylation of the hTERT promoter to evade CTCF-mediated repression near the proximal exon region of hTERT [340, 341]. However, in certain cancers, such as ovarian and testicular cancers, promoter methylation occurs infrequently, maintaining levels similar to those in normal tissues [342]. TERT promoter mutations are also rare in ovarian cancer. Among ovarian cancer subtypes, these mutations are most frequently observed in ovarian clear cell carcinoma and adult granulosa cell tumor; however, only 15.9% and 22% of patients with these respective subtypes exhibit promoter mutations [343, 344]. In these tissues, tumors may rely on mechanisms involving the Brother of the Regulator of Imprinted Sites (BORIS) to activate telomerase and circumvent CTCF-mediated repression, thus ensuring the unrestricted proliferation of cancer cells [340]. Originally discovered in the testes, BORIS is the paralogue of the chromatin factor encoded by CTCF [21, 345]. BORIS maintains a relative balance with CTCF, and by occupying CTCF binding sites, it facilitates hTERT transcriptional activation in ovarian and testicular cancers [340]. In advanced ovarian cancer, TERT expression is positively correlated with sensitivity to platinum-based chemotherapy. Among patients who achieved a complete response, 74% exhibited high TERT expression, whereas 66% of those with partial or no response had low TERT expression [346]. Additionally, TA can aid in staging, as stage IV tumors exhibit higher TA levels compared to tumors at stage II or below [347]. Generally speaking, longer LTL before diagnosis may increase the risk of the ovaries [348]. However, the LTL after diagnosis cannot fully predict prognosis [349]. In malignant tissues, telomere length does not differ significantly among high-grade and low-grade serous carcinomas and low-grade endometrioid carcinoma [350]. However, the mean telomere length in ovarian clear cell carcinoma is substantially longer than in these three subtypes, which may partially explain its chemoresistance [350, 351]. Compared to endometrioid carcinoma, which also originates from endometriosis, ovarian clear cell carcinoma is associated with a notably higher risk of mortality and distinct molecular mechanisms [352, 353]. Additionally, compared to serous ovarian carcinoma, ovarian clear cell carcinoma has a relatively lower 5-year survival rate, with more frequent solid organ involvement and lymph node metastasis upon recurrence [351]. Telomerase activation may also be associated with an increased risk of mortality. Furthermore, patients with ovarian clear cell carcinoma exhibit significant telomere heterogeneity, with subpopulations displaying longer telomeres facing a higher risk of mortality [350]. These findings may suggest the presence of distinct subgroups within this subtype, warranting further investigation.

#### Thyroid cancer

Thyroid cancer is the most prevalent malignancy within the endocrine system [354]. Longer telomeres are associated with an increased risk of thyroid cancer; however, most thyroid cancer patients exhibit significantly shorter telomeres compared to healthy individuals [355–357]. Tumors with extensive TERT promoter methylation and aggressive thyroid cancers often exhibit shorter telomeres. This shortening may induce a more relaxed conformation at the 5p chromosomal terminus, closely associated with TERT, facilitating increased transcriptional activity and ultimately contributing to a poorer prognosis [358]. Over 95% of thyroid cancer cases originate from follicular thyroid cells (thyrocytes). The primary subtypes include papillary thyroid carcinoma (PTC), accounting for approximately 85%; follicular thyroid carcinoma (FTC), 10-15%; poorly differentiated thyroid carcinoma (PDTC), 5-10%; and anaplastic thyroid carcinoma (ATC), 2-3% [359]. The TERT promoter mutation frequencies for PTC, FTC, PDTC, and ATC are 12%, 14%, 38%, and 46%, respectively [360]. ATC is a rare malignant tumor, with the most common gene mutation being the TERT promoter mutation [361]. NGS analysis reveals a promoter mutation rate of up to 73% in ATC [362]. Telomerase activation is absent in normal thyroid tissue, and its activation frequency in thyroid cancer is considerably lower compared to other cancers. Among thyroid cancer subtypes, the frequency of telomerase positivity is 20% in PTC, whereas FTC and ATC show higher positivity rates, reaching 66% [363]. This activation of telomerase may underlie the increased aggressiveness observed in these subtypes [363]. In most patients, particularly those with papillary thyroid carcinoma (PTC) and follicular thyroid carcinoma (FTC), low telomerase activity may be an intrinsic factor contributing to the typically indolent clinical course of thyroid cancer and its overall low mortality rate [364]. The expression of telomerase activity may be associated with high methylation levels upstream of the transcription start site (UTSS), where increased UTSS methylation can prevent repressor protein binding, thereby enhancing TERT expression [355]. Two cell lines, FTC-133 and FTC-238, derived from metastatic FTC patients, demonstrate this phenomenon. The latter, exhibiting greater malignancy, shows higher UTSS methylation levels and elevated TERT expression compared to FTC-133 [365]. Clinically, aggressive thyroid tumors also display significantly elevated UTSS methylation [358].

#### Liver cancer

Globally, the occurrence and progression of liver cancer are linked to hepatitis B(HBV) and C(HCV) viruses infections in approximately 80% of cases, with additional associations with alcohol consumption and exposure to aflatoxin B1 [366–368]. Hepatocellular carcinoma (HCC) comprises around 90% of liver cancers, intrahepatic Cholangiocarcinoma(ICC) accounts for 10%, and combined hepatocellular-cIntrahepatic cholangiocarcinoma(cHCC-ICC) makes up about 4% [369]. In the normal liver, a small subset of hepatocytes exhibit elevated telomerase expression, contributing to tissue repair following liver injury [370]. In general, telomeres in normal biliary epithelial cells are longer than those in hepatocytes [371]. In non-diseased hepatic and biliary cells, age-related shortening of telomeres appears to be minimal [372]. The frequency of telomerase activation in both HCC and ICC is approximately 80–85%, suggesting minimal variation in telomerase reactivation across different liver cancer subtypes [373, 374]. In HCC, elevated telomerase activity, TERT overexpression, and longer telomeres are often indicative of a poor prognosis. Extended telomere length is also associated with poorer tumor differentiation and stem cell-like characteristics [214, 375, 376]. The association between LTL and the risk of HCC or ICC follows an inverted "J" pattern, where shorter telomeres correlate with an increased risk of disease [377]. In HCC, different etiological factors can lead to varying changes in promoter activity. HBV DNA can integrate into or upstream of the TERT gene, thus bypassing the need for promoter mutations to activate telomerase expression [378]. Globally, the rate of TERT promoter mutations in HCV-associated HCC cases are generally higher than in HBV-associated cases. For example, in Asia, the overall TERT promoter mutation frequency is 28.9% in HBV-positive HCC, 69.7% in HCV-related cases, and 52.6% in non-viral HCC. In Europe, hotspot mutation rates are 61.5%, 57.7%, and 42.7% in HCV-related, non-viral, and HBV-associated HCC, respectively [379]. Although ethnic or regional factors may contribute to differences in promoter mutation rates across subtypes, these data indicate that HBV-associated HCC has the lowest promoter mutation rate overall, followed by non-viral HCC, with HCV-associated HCC exhibiting the highest frequency. Promoter mutations in hepatocellular carcinoma (HCC) commonly occur upstream of the ATG translation start site at positions − 124 (G > A) and − 146 (G > A) [380, 381]. Additionally, approximately 7.5% of mutations are found at position − 297 (C > T) [381]. The former constitutes the vast majority of mutations [369]. In intrahepatic cholangiocarcinoma (ICC), TERT promoter mutations are present in only 5% of cases, with mutation rates falling below 1% in certain populations [382, 383]. In contrast, combined hepatocellular-cholangiocarcinoma (HCC-ICC) may exhibit a TERT promoter mutation rate as high as 53.3%, likely influenced by the presence of HCC [383].

#### Ohter cancers

Beyond the heterogeneity observed in telomerase and telomere dynamics as biomarkers across the aforementioned nine cancer subtypes, other cancers also exhibit similar patterns of diversity. From an organizational standpoint, in colorectal cancer, a longer LTL is associated with a significantly increased risk of rectal cancer within seven years after the blood draw, whereas this association does not reach significance in colon cancer [384]. TA assays indicate that telomerase activity is significantly higher in colon cancer than in rectal cancer. Further subdividing the colon into the right colon—comprising the cecum, ascending colon, and transverse colon—and the left colon—consisting of the splenic flexure, descending colon, and sigmoid colon—reveals that telomerase activity is also significantly higher in the right colon than in the left colon [385]. These findings indicate that, from the perspective of telomerase activity and telomere dynamics, there is biomarker-based evidence supporting a tripartite division of colorectal cancer for diagnostic and therapeutic purposes, grounded in distinct structural, physiological, and pathological environments [386]. In addition, as there remains controversy regarding whether normal colonic tissue expresses telomerase, further research is needed to clarify this issue [385, 387]. From the perspective of genetic mutations, prostate cancer generally lacks TERT promoter mutations, precluding their use as biomarkers [115]. However, other mutations may contribute to telomerase activation and could serve as alternative biomarkers. In normal human prostate epithelial cells, the androgen receptor (AR) binds to the TERT promoter and collaborates with the tumor suppressor p53 to repress TERT expression, thereby rendering telomerase inactive [388]. Similarly, in normal prostate tissue from both mice and rhesus monkeys, telomerase activity is absent [389, 390]. However, following castration, detectable telomerase activity emerges in these animals' prostate tissue, and the reintroduction of androgen suppresses telomerase activity once more, suggesting an inhibitory role of androgen on telomerase. In contrast, in prostate cancer cells harboring mutated AR, androgen stimulates TERT expression, indicating that androgen in conjunction with a mutated AR may promote telomerase activity within prostate cancer cells [388]. This suggests that focusing on telomerase in the context of genetic mutations may yield indirect biomarkers. From the perspective of biomarker acquisition, utilizing urine as a diagnostic sample could facilitate the broader application of telomerase as a biomarker for cancer diagnosis. Urothelial carcinomas (UCs) are classified into two primary subtypes: bladder urothelial carcinoma (UCB), which comprises approximately 90% of all UC cases, and upper tract urothelial carcinoma (UTUC), which includes renal pelvis and ureteral UC (UCRP and UCU), accounting for the remaining 10% [391]. TERT promoter mutations are present in less than 20% of UCU, in 45% of UCRP, and in approximately 85% of UCB [392]. The LTL appears to have a relatively weak association with bladder cancer risk [393]. Remarkably, TERT promoter mutations can be detected in the urine of patients with UCB up to a decade prior to diagnosis [394]. In the majority of patients with UCB, TERT promoter mutations in urine rapidly disappear following tumor resection [395]. However, in a subset of patients, these mutations persist, strongly indicating a risk of recurrence [395]. Thus, TERT promoter mutations may serve as a biomarker for bladder cancer relapse, enabling periodic urine-based monitoring to ensure timely intervention and management.

## Cancer treatment targeting telomeres and telomerase

Because telomeres and telomerase ensure the continuous replication of cancer cells, the treatment plan targeting telomerase has sufficient research value [3]. Since telomeres and telomerase ensure the continuous replication of cancer cells, targeting telomerase for therapeutic intervention holds significant research value. Currently, telomerase-targeted therapies primarily consist of immunotherapies and telomerase inhibitors. Immunotherapies include oncolytic viruses and vaccines. Telomerase inhibitors are classified into two categories: direct inhibitors, including oligonucleotide inhibitors and natural or artificial compound inhibitors, and indirect inhibitors, such as G-quadruplex stabilizers and nucleoside analogues. However, currently, only oligonucleotide inhibitors and natural or artificial compound inhibitors are being applied in clinical research. In clinical practice, adverse toxicities often render continued treatment intolerable for patients, while discontinuation of therapy risks the reactivation of telomerase, presenting a conundrum that remains unresolved [396]. Most of them are still in cell and animal experiments, with only a small portion have undergone clinical trials [397]. In vitro experiments reveal that candidate drugs frequently require as many as 40 cell doublings to elicit observable effects such as apoptosis or senescence [396].

### Immunotherapy

#### Oncolytic virus

Oncolytic viruses are viruses that can selectively replicate within cancer cells, producing a therapeutic effect [398]. The reactivation of telomerase is prevalent in cancer cells. Therefore, researchers have designed oncolytic viruses targeting telomerase for clinical application, such as KH901, Telomelysin, and OB-301 [399–401]. OBP-301 is a modified adenovirus. Treatment trials involving 13 esophageal cancer patients who were not candidates for surgery or chemotherapy, and 20 patients with advanced HCC who had no applicable treatment options, showed that OBP-301 had good tolerability, increased local CD8 + T-cell counts, and demonstrated local tumor control. However, the systemic antitumor effects were not significant [401, 402]. Telomelysin is also a modified adenovirus. Studies have shown that, after intratumoral injection of the virus, it is also present in distant untreated tumors. Following treatment in 16 patients, 7 patients exhibited a therapeutic response, indicating the potential for systemic antitumor activity. Overall, Telomelysin has shown good tolerability [402]. KH901, when administered to 13 patients with head and neck cancer, increased levels of TNF, IL-6, and IL-10 [399]. However, due to the small sample size, its efficacy requires further validation. Similar to the previous two oncolytic viruses, KH901 has shown good tolerability, with side effects such as pain following intratumoral injection being manageable [399]. Beyond the oncolytic viruses currently in clinical trials, there are several others with therapeutic potential. OBP-502 is a variant of OBP-301 that expresses RGD peptides in the E3 region, enabling interaction with integrin αvβ5 expressed on tumor cells, thereby facilitating viral entry more efficiently than OBP-301 [403]. It leads to the recruitment of CD8 + lymphocytes and inhibits the infiltration of Foxp3-positive lymphocytes into tumors, promoting autophagy and apoptosis in tumor cells, inhibiting tumor growth, and possessing the potential to convert ‘cold’ tumors into ‘hot’ tumors [403]. OBP-702, on the other hand, incorporates a function driven by the hTERT promoter, enhancing the expression of the p53 gene in tumor cells, potentially conferring stronger antitumor effects compared to OBP-301 [404].

#### Tumor vaccine

TERT peptide vaccines address one of the key issues in immuno-oncology by converting non-immunogenic tumors into immunogenic ones, thereby activating the host's immune response. In clinical applications, some vaccines used in clinical trials include CpG oligodeoxynucleotides (CpG-ODN) that mature plasmacytoid dendritic cells (PDC) into potent antigen-presenting cells, and those that stimulate cytotoxic T lymphocytes (CTLs) to recognize the tumor antigen TERT_572Y_. While these approaches help to improve the anti-tumor microenvironment, they have not demonstrated anti-tumor efficacy [405, 406]. They may be more suitable as adjuvant treatment options. In a Phase II clinical trial for the treatment of NSCLC with vx-001, the mean OS of 89 patients in the treatment group did not exceed that of 101 patients in the placebo group. This outcome is attributed to the fact that only 29% of patients in the treatment group developed a durable TERT-specific immune response. Patients who generated this response had an OS of 21.3 months, compared to 13.4 months for those who did not, demonstrating a statistically significant difference [407]. Studies of GV1001 have shown that, whether used alone in pancreatic cancer or in combination with HR2822 for NSCLC, it can induce T-cell responses and demonstrate extended OS [408, 409]. However, similar outcomes were not observed in liver cancer, suggesting that further investigation into the underlying mechanisms is warranted [410]. Another vaccine, known as UV1, has shown promising results in clinical trials. Administered to 52 patients with melanoma, NSCLC, and prostate cancer, UV1 demonstrated excellent safety and tolerability [411–414]. The vaccine can induce multifunctional CD4 + Th1 cells, and the immune response (IR) elicited by UV1 was associated with prolonged survival in patients with these three different types of cancer, warranting further development and investigation [413]. The GX301 vaccine consists of four peptides (540–548, 611–626, 672–686, and 766–780) and two adjuvants, Montanide ISA-51 VG and imiquimod. In a cohort of 63 evaluable patients with prostate cancer, the administration regimen was found to be safe and effectively induced an immune response in more than 95% of patients, affecting the proportions of Treg cells and other T-cell subsets. However, its impact on OS in patients requires validation in future studies [415]. INO-1400 and INO-1401 are synthetic DNA plasmids encoding modified human telomerase proteins. INO-9012 is a DNA plasmid encoding recombinant human IL-12 (p35 and p40 subunits) [416]. INO-1400, INO-1401, and INO-9012 are vaccines composed of plasmids encoding the catalytic subunit of the tumor-associated antigen hTERT [416]. Treatment with INO-1400 or INO-1401, either alone or in combination with INO-9012, in 93 patients with at least one of nine specific solid tumors, demonstrated acceptable safety and elicited an immune response in 96% of patients. These treatments promoted hTERT-specific IFN-γ production, generated hTERT-specific CD4 + and CD8 + T cells expressing CD38, and induced hTERT-specific activated CD8 + CTLs expressing perforin and granzymes [416]. It is important to note that these treatments are often administered in the advanced stages of cancer, and their effects during the early stages may require further investigation.

### Telomerase inhibitor

#### Direct inhibitors

##### Oligonucleotide

Among oligonucleotide inhibitors, the only drug currently in clinical trials is Imetelstat (GRN163L), a 13-mer oligonucleotide N3' → P5'-thio-phosphoramidate lipid conjugate [417]. Imetelstat consists of 13 nucleotides linked by phosphorothioate amidate bonds, which bind to the template region of telomerase, thereby preventing its interaction with telomeres and inhibiting telomerase function. In a trial involving 39 children with recurrent or refractory central nervous system (CNS) malignancies, the most common Grade 3/4 toxicities associated with Imetelstat treatment included thrombocytopenia, lymphopenia, and neutropenia [418]. The trial was prematurely terminated due to intratumoral hemorrhage caused by thrombocytopenia in two children. Imetelstat inhibits TA in peripheral blood mononuclear cells (PBMCs) for 8 days, and its thrombocytopenic effect precludes more frequent dosing than every 8 days in children with CNS tumors [418]. This justifies an every-eight-day dosing schedule. While the study did not assess telomere shortening, TA within a tumor was found to be suppressed by more than 95% at 26.5 h post-administration in one patient [418]. Therefore, while Imetelstat inhibited TA to some extent in the trial, it was insufficient to inhibit tumor cell proliferation [418]. In a trial involving 96 adult patients with NSCLC, the dose-limiting myelosuppressive effects of Imetelstat in combination with chemotherapy were pronounced, which led to the premature termination of the trial [419]. The dosing schedule was every eight days, and the outcome measures did not include evaluations of TA or telomere length. However, based on the available results, there was a trend toward increased OS when Imetelstat was combined with chemotherapy, particularly in patients with shorter telomeres, compared to chemotherapy alone. Thus, for non-neurological tumors, there may still be clinical potential for using Imetelstat alone, or in combination with treatments that reduce bleeding and increase platelet counts, which could broaden its application prospects. In both trials, no shortening of telomeres was observed in the subjects, suggesting that the mechanism of action for Imetelstat may not be through telomerase inhibition but rather through sequence-independent immunostimulatory effects of the phosphoramidates [420].

T-oligos, which share homology with the 3' overhangs of mammalian chromosomes, have demonstrated anticancer activity both in vitro and in vivo across various cancer types [421]. T-oligos exhibit multiple anticancer mechanisms related to telomeres and telomerase. With respect to telomeres, T-oligos may shorten telomeres in cancer cells by disrupting shelterin proteins, leading to cellular senescence and apoptosis [421]. T-oligos may also involve c-Jun N-terminal kinase (JNK)-mediated reduction of TERT, thereby decreases TA [422]. T-oligos may even stabilize G-quadruplex structures, thereby inhibiting TA [422]. Additionally, T-oligos promote autophagy in tumor cells and inhibit angiogenesis [423, 424]. Unfortunately, they face the challenge of rapid degradation by nucleases [421]. To address this, researchers have co-administered T-oligos with the α-helical cationic peptide PVBLG-8 (PVBLG). In animal models, this approach reduced tumor volume, thereby broadening the prospects for the clinical application of T-oligos [425].

#### Natural or artificial compounds

Currently, there are over two hundred natural or synthetically derived telomerase inhibitors. Among Natural or artificial compounds, MST-312 is a telomerase inhibitor derived from the natural compound epigallocatechin gallate (EGCG). EGCG is the major catechin in green tea and exhibits anticancer properties, including effective inhibition of telomerase [426]. MST-312 shows more pronounced inhibition of telomerase compared to EGCG [427]. It inhibits TA by suppressing the expression of proteins in the Shelterin complex and inducing telomere dysfunction [428]. In a clinical comparative study involving 73 patients with multiple myeloma following stem cell transplantation, patients treated with MST-312 showed a significant difference in progression-free survival (PFS) during the first and second years post-treatment. Additionally, there was a significant reduction in IL-6 and TNF-α gene expression, suggesting that MST-312 not only inhibits telomerase function but also exhibits antitumor activity. It is noteworthy that out of the original 35 patients in the treatment group, 12 patients were switched to the control group due to intolerance, indicating that there may be limitations to its therapeutic efficacy and that further investigation into the specific mechanisms is warranted [429]. These clinical trials did not assess the impact on normal cells. However, in vitro experiments comparing ovarian cancer cells with normal ovarian surface epithelial (OSE) cells revealed that MST-312 exhibited dose-dependent cytotoxicity toward cancer cells [430]. However, MST-312 showed no toxicity to OSE cells at concentrations below 5 μM and exhibited a protective effect at lower doses, while higher doses had an inhibitory effect on OSE cells [430]. The safety of this concentration for normal cells was also validated in human immortalized normal breast epithelial cells (MCF-10A) [431].

Perifosine is an orally administered alkylphospholipid that has been used in Phase III clinical trials for multiple myeloma. Studies have shown that one of its mechanisms of action involves modulation of AKT and telomerase [432, 433]. AKT participates in the synthesis of telomerase, and the AKT inhibitor perifosine has been shown to reduce telomere length and cellular viability [433]. This effect has been validated in multiple cancer cell lines. In a xenograft model of breast cancer, perifosine reduced tumor volume. In a clinical trial involving six patients, four patients with CLL exhibited decreased TA, and two of these patients experienced shortening of their telomeres [432].

BIBR1532 is a non-peptidic, non-nucleoside small molecule compound that acts as a direct telomerase inhibitor by specifically binding to the active site of hTERT, thereby preventing its interaction with other components of the telomerase complex [434]. BIBR1532 is considered to possess potential antitumor activity because it can induce replicative senescence and apoptosis in various cancers [435, 436]. BIBR1532 exerts its effects by transcriptionally inhibiting survivin-mediated expression of c-Myc and hTERT, leading to a decrease in TA. It also possesses telomere-independent functions, such as increasing the expression of p73 and p21, upregulating the Bax/Bcl-2 molecular ratio, and enhancing P53 expression, ultimately resulting in the induction of tumor cell apoptosis [437, 438]. Additionally, BIBR1532 downregulates EGFR, the ratio of phosphorylated ERK to total ERK, and matrix metalloproteinases (MMP)-1, -2, and -9, thereby inhibiting cell growth, migration, and invasion [436]. BIBR1532 can also significantly enhance the ability of radiotherapy to produce dsDNA and promote the release of mtDNA induced by ferroptosis-related mitochondrial stress [439]. This may enhance the clinical research prospects of BIBR1532, but before clinical trials, its poor pharmacokinetic properties remain a challenge [3].

Costunolide, a natural sesquiterpene compound, possesses anticancer activity and has been found to exhibit telomerase inhibitory effects [440]. Costunolide, by inhibiting telomerase, reduces the concentration of the serum HCC diagnostic marker alpha-fetoprotein (AFP), thereby suppressing the proliferation of HCC cells [441]. ROS and mitochondrial function are considered effective mechanisms for enhancing antitelomerase therapy [442]. The telomerase inhibitor costunolide induces apoptosis in glioma cells via a ROS-dependent pathway [442].

Curcumin (diferuloylmethane), the principal yellow-colored dietary pigment derived from the rhizomes of turmeric (*Curcuma longa* L.), has demonstrated anticancer properties in various cellular experiments and animal models [443–445]. Curcumin acts as a telomerase inhibitor by suppressing TERT expression in cancer cells, thereby inhibiting TA [445]. Importantly, no significant toxic side effects have been observed in studies, enhancing its potential as a therapeutic agent [445]. Martí-Centelles et al. capitalized on the telomerase-inhibiting properties of curcumin to design pyrazole moieties into curcuminoid structures [446]. The optimal compound identified was (E)-3,5-bis [β-(4-hydroxy-3-methoxyphenyl)ethenyl]-1H-pyrazole, which effectively reduced hTERT expression [446]. Notably, this novel compound also suppressed c-Myc expression, an effect not observed with curcumin alone [445, 446].

Boldine (1,10-dimethoxy-2,9-dihydroxyaporphine) is an aporphine alkaloid derived from the bark of boldo trees (*Peumus boldus*) and *Lindera aggregata*. It possesses hepatoprotective, cytoprotective, antipyretic, and anti-inflammatory properties [447–449]. In cancer therapy, Boldine inhibits cancer cell viability and promotes apoptosis through the inactivation of AKT and activation of glycogen synthase kinase-3β (GSK-3β) [450, 451]. Additionally, Boldine inhibits TA by forming two hydrogen bonds with the amide hydrogen of Gln190. Kazemi Noureini S discovered that its derivative BSB (N-benzylsecoboldine) has similar effects and may act as a noncompetitive inhibitor by interfering with substrate-enzyme interactions. However, the study utilized castaneum TERT, and further validation is required using hTERT [452].

Many antibiotics also possess antitumor properties and can function as telomerase inhibitors. For instance, chrolactomycin, UCS1025A, and radicicol can be extracted from *Streptomyces* species 569N-3, *Streptomyces fradiae*, *Acremonium* species, and *Monosporium bonorden*, respectively [453–455]. Betori et al. developed a chrolactomycin analogue named Chrolog and subsequently optimized it to create the derivative NU-1 [456, 457]. NU-1 is capable of long-term inhibition of TERT in tumor cells, which may be essential for force telomere erosion [457]. However, it also affects the proliferation of stem cells and progenitor cells in normal tissues. In a study using BALB/c mice with CT26 colorectal tumors implanted subcutaneously, NU-1 was shown to enhance the efficacy of radiotherapy through DNA damage response pathways, potentially leading to tumor elimination [457]. However, short-term monotherapy with NU-1 was neither toxic nor effective [457].

U-73122 was synthesized as an amphiphilic alkylating agent [458]. The N-substituted side chain and/or group of U-73122 also plays a role in the inhibitory action of these maleimides on telomerase and promotes apoptosis in Jurkat and HL60 cells [458].

Fletcher TM et al. synthesized a series of quinoline-imidazole hybrid compounds [459]. Among these, phenyl and dimethylamino substituted quinazoline-imidazole derivatives exhibited optimal telomerase inhibitory and antitumor activities in the U251, HeLa, HepG-2, and A549 cancer cells, suggesting their potential as anticancer agents [459].

Juan S et al. synthesized a series of 5-(quinolin-2-yl)-1,3,4-oxadiazole-2(3H)-thione quinoline derivatives and evaluated their telomerase inhibitory and antitumor activities in HepG2, SGC-7901, and MCF-7 cell lines [460]. The results indicated that two compounds, featuring a fluoro substituent at the ortho position, and a chloro substituent at the para position of aniline moieties, hold the greatest potential for further investigation [460].

Hayakawa et al. reported on 2- [3-(trifluoromethyl)phenyl]isothiazolin-3-one (TMPI), which inhibits partially purified telomerase extracted from AH7974 rat hepatoma cells by targeting cysteine residues [461].

Culletta et al. conducted in silico modeling and virtual screening of hundreds of arylsulfonamides, subsequently identifying N- [4-(3,5-dimethylpyrazol-1-yl)phenyl]-4-aminobenzenesulfonamide as the most promising telomerase inhibitor in vitro cellular assays [462]. This compound was involved in key interactions with residues at the active site, including Arg557, Ile550, and Gly553 [462]. Notably, it exhibited greater inhibitory effects on HCT-116 and MCF7 cell lines but showed less efficacy against the K-562 cell line [462].

Shi et al. developed a series of benzophenone and phenstatin derivatives. Among these, the compound 2-methoxy-5-(2-methoxybenzoyl)phenyl((2S,5R)-5-(5-methyl-2,4-dioxo-3,4-dihydropyrimidin-1(2H)-yl)-2,5-dihydrofuran-2-yl)methyl succinate exhibited significant inhibitory activity against telomerase [463]. Additionally, this compound may induce apoptosis in SGC-7901 cells through the dissipation of mitochondrial membrane potential (MMP). Furthermore, it was found to suppress tumor growth in S180 and HepG2 xenograft mouse models [463].

Xue W et al. designed novel myricetin (43) derivatives. Among these, the most potent telomerase inhibitor identified was 2-(5,7-dimethoxy-4-oxo-2-(3,4,5-trimethoxyphenyl)-4H-chromen-3-yloxy)-N'-(2-furoyl)hydrazine [464]. This compound was found to reduce the expression of p65 and TERT proteins and inhibit the proliferation of MDA-MB-231 breast cancer cells [464].

Liu et al. developed a series of novel 2-chloro-pyridine derivatives incorporating flavone, chromone, or dihydropyrazole units [465]. Among these, the compounds capable of binding to the active site of telomerase (PDB code: 3DU6) demonstrated the strongest inhibitory effects on both TA and cellular proliferation in SGC-7901 gastric cancer cells [465]. Furthermore, the team designed novel coumarin derivatives containing a 4,5-dihydropyrazole moiety, novel N-phenylacetyl (sulfonyl) 4,5-dihydropyrazole derivatives, and 3-(2-(3-methyl-5-substituted-phenyl-4,5-dihydropyrazol-1-yl)-2-oxo-ethoxy)-2-substituted-phenyl-4H-chromen-4-one derivatives, all targeted to the active site of telomerase (PDB code: 3DU6) [466–468]. Lu et al. also designed novel 2-methyl-4,5-substituted benzo [f]-3,3a,4,5-tetrahydro-pyrazolo [1,5-d] [1, 4]oxazepin-8(7H)-one derivatives and found that these compounds inhibit the proliferation of MGC-803 cancer cells by suppressing the expression of Cyclin D1, TERT, phospho-AKT, and the PI3K/AKT pathway [469]. Other members of this team have also published research based on the 3DU6 active site. In the research conducted by Luo et al., a series of novel aryl-2H-pyrazole derivatives bearing oxygen-containing heterocyclic groups was investigated. Shi JB’s study focused on a series of novel pyrazole-5-carboxamide and pyrazolo [pyrimidine] derivatives. Additionally, Zhang F et al. developed a series of new 1,3,4-oxadiazole derivatives (6a-6x) containing pyridine and acylhydrazone moieties. Among these, the compounds that target the 3DU6 active site were identified as the most promising candidates [470–472]. They validated that within these series of compounds, those targeting the 3DU6 site were the most effective in inhibiting the activity of cancer cells and telomerase.

Fan et al. investigated two series of flavonoid derivatives, among which 7-O-((1-(2,3,4-tri-O-acetyl-α-L-arabinopyranosyl)-1H-1,2,3-triazol-4-yl)methyl)-3,5,4′-tri-O-acetyl-kaempferol exhibited the most potent anticancer and telomerase inhibitory effects in A549, HepG2, HeLa, MGC-803, and SGC-7901 cell lines [473]. This efficacy may be attributed to the conserved residues Lys 437 and Asn 421, which facilitate ligand binding through hydrogen bonding interactions [473].

Xiao X et al. developed a series of compounds centered around an N-acyl-4,5-dihydropyrazole core, highlighting the importance of the N-sulfonyl moiety for telomerase inhibition. This research, together with the aforementioned studies, may collectively validate the potential of utilizing the 4,5-dihydropyrazole scaffold in the development of telomerase inhibitors [466, 466, 474].

Han et al. designed a series of new 2-phenyl-4H-chromone derivatives containing a 1,3,4-oxadiazole moiety, targeting dyskerin. Among these, (E)-2-(5,7-dimethoxy-4-oxo-2-(3,4,5-trimethoxyphenyl)-4H-chromen-3-yloxy)-N-(5-styryl-1,3,4-oxadiazol-2-yl)acetamide demonstrated the most significant reduction in dyskerin levels in MGC-803 cells [456]. This compound also reduced the expression of NHP2 and NOP10 within the dyskerin-NHP2-NOP10 trimer, thereby inhibiting TA. It was also the most effective compound in this series for inhibiting cell proliferation and promoting apoptosis.

Resveratrol (3,5,4’ trihydroxystilbene), is a phytoalexin phenolic compound [475]. Resveratrol is contained in the root of *Polygonumcuspidatum* plant, as well as peanut and small soft fruits. It has also been found to act as an inhibitors of telomerase activity, demonstrating significant activity against colorectal cancer, breast cancer, liver cancer, melanom [476–479]. Inducing cell cycle arrest in the S phase, resveratrol effectively inhibits the proliferation of MCF-7 breast cancer cells and inhibits telomerase-associated apoptosis and senescenc [477]. Additionally, resveratrol combined with chemotherapeutic agents directly or indirectly interferes with telomerase activity and inhibits hTERT protein expression, affecting signaling pathways including apoptosis and cell cycle control, such as STAT3, Akt, SIRT1/Nrf2, suggesting that its enhanced antitelomerase activity mainly mediates anticancer effects of resveratrol, reduced hTERT mRNA expression, and highlighting its potential as a cancer therapy [480, 481]. Rahimifard et al. intervened gastric cancer cells through the combination of resveratrol and cisplatin and found that it induced G0/G1 cell cycle arrest, reduced telomerase activity, inhibited metastasis, induced apoptosis and cellular senescence through the P38/P53 and P16/P21 pathways and played a synergistic effect [482].

Quercetin (3,5,7,3',4'-pentahidroksiflavon) is a polyphenolic compound synthesized as a biologically active secondary metabolite, Quercetin is a flavonoid that the main sources of which are fruits and vegetables, propolis, especially tea, apples (4.01 mg/100 g), broccoli (13.7 mg/100 g) and onions (45 mg/100 g) as well as red wine (3.16 mg/100 g), includes large amounts of a flavonol called quercetin glycosides [483]. It synergistically produces an anti-telomerase effect by modulating DNA damage, down-regulating hTERT to induce apoptosis and decreasing telomerase activity, decreasing the expression of p53 and p21-rasoncogene, and inhibiting the activity of Hsp90. Quercetin also induces cell cycle arrest in the G1 phase of the cell cycle, leading to a decrease in Akt phosphorylation, synergistically reducing senescence, showing significant activity against leukemia, ovarian, breast, laryngeal, nasopharyngeal, colon, and gliomas [430, 484, 485].

Tannins (or gallotannin) are polyphenolic compounds with molecular weight of 500–3000 Dalton [486]. Tannic acid is a naturally occurring compound that is contained in tea, coffee, grapes, red wine, beans, and nuts such as hazelnut and most fruits and vegetables. It inhibited DNA polymerases, proteasome, and poly (ADP-ribose) glycopyrrolate, and induced telomere shorting by inhibiting telomerase leading to cellular senescence [484, 487]. Tannic acid possesses antioxidant or prooxidant properties, depending on cell type and concentration [488]. Cosan et al. found that tannins exhibited stronger inhibition of telomerase activity and promotion of apoptosis in breast and rectal adenocarcinomas compared to resveratrol.

Dogan et al. found Rapamycin, an mTOR inhibitor, suppressed telomerase activity at translational and posttranslational levels, inducing apoptosis. Targeting CSCs, Rapamycin disrupted self-renewal, synergistically affecting mTOR and telomerase-mediated cancer, counteracting carcinogenesis [489]. Telomerase is a complex consisting of an RNA template (hTR) and the catalytic protein hTERT, which possesses reverse transcriptase activity. The expression of hTERT serves as the rate-limiting factor for human telomerase activity and is considered a sensitive marker for telomerase function. Notably, the mTOR signaling pathway may play a significant role in the regulation of telomerase activity. Bae-Jump et al. conducted real-time quantification of hTERT mRNA expression in ovarian and cervical cancer cell lines using quantitative RT-PCR. Treatment with rapamycin at a concentration of 20 nM resulted in a significant reduction in hTERT mRNA levels across all cervical and ovarian cancer cell lines. This reduction was observed in the rapamycin-resistant ovarian cancer cell line HEY, as well as in all other ovarian and cervical cancer cell lines sensitive to rapamycin. These findings suggest that rapamycin may inhibit telomerase activity through the rapid reduction of hTERT mRNA levels. Furthermore, rapamycin demonstrated a dose-dependent inhibition of cancer cell line growth, with an IC50 value of less than 50 nM [490].

### Indirect inhibitors

#### Nucleoside analogues

Nucleoside analogs capitalize on the elevated TA in cancer cells by incorporating into the telomeres, thereby selectively hindering the binding of shelterin complexes to telomeric DNA and inducing genomic instability in telomerase-positive cells, ultimately accelerating cell death [3, 491].

The anticancer agent 5-fluoro-2'-deoxyuridine(5-FdU), which has received FDA approval and is commonly used in the treatment of colorectal and metastatic liver cancers, has recently been found to exert its mechanisms of action through telomeres and telomerase. The substitution of four thymidine residues with 5-FdU in telomeres results in a more than 20-fold reduction in the binding affinity of POT1-TPP1 to DNA, leading to its displacement by the RPA protein. This subsequently activates ATR kinase, triggers Chk1 phosphorylation, and induces p53 activation, promoting rapid apoptosis in tumor cells at clinically feasible drug concentrations [491].

6-thio-2-deoxyguanosine (6-Thio-dG) is another nucleoside analog that, when incorporated into the telomeric DNA of telomerase-positive cells, leads to telomere dysfunction, genomic instability, and cell death. In vivo experiments have shown that 6-thio-dG increases telomere damage, induces cell cycle arrest and apoptosis, and reduces proliferation in tumor cells within mice [492]. However, issues related to its low therapeutic efficacy and dose-limiting toxic side effects must be resolved before expanding clinical research [493].

Azidothymidine (AZT) is a thymidine analog originally used for the treatment of human immunodeficiency virus (HIV) [494, 495]. AZT, the first reported telomerase inhibitor, holds significant historical importance [496]. Given the structural similarity between the catalytic core of telomerase reverse transcriptase (TERT) and HIV-1 reverse transcriptase (RT), AZT also acts as a telomerase inhibitor [497]. In vitro studies have demonstrated that AZT not only suppresses telomerase activity by downregulating TERT expression but also exerts non-telomeric effects of TERT. Specifically, it significantly reduces the expression levels of C-Myc and Cyclin D1, thereby inhibiting the invasion of cancer cells [496]. Given the extended time required for AZT to exert its therapeutic effects, its application as an adjuvant therapy to reduce tumor volume or as a postoperative treatment for patients without metastasis demonstrates considerable clinical potential [498]. However, in the treatment of advanced-stage cancer, the delayed onset of AZT’s efficacy may result in disease progression before the drug’s benefits can be realized, thereby limiting its clinical utility in this setting. This presents a notable clinical challenge in its use. As a telomerase inhibitor, it was evaluated in Phase II clinical trials. Although AZT demonstrated some efficacy in tumors such as rectal and pancreatic cancers, its mechanism also involves inhibition normal cellular DNA polymerases, which has limited its further clinical development [499–504].

5-Methylcarboxyindolyl-2'-deoxyriboside 5'-triphosphate (5-MeCITP) is an indole nucleoside analog that, through a mechanism similar to that of AZT, effectively reduces TA in cell lines such as HCT116, MIA PaCa-2, A549, HeLa, WI-38, and U2OS [497]. Its efficacy is comparable to that of AZT; however, it is better tolerated by normal cells, thus potentially offering greater promise as a telomerase inhibitor for cancer therapy [497].

Furthermore, several nucleoside analogs have been identified as potential human telomerase inhibitors and are considered promising candidates for cancer therapy applications. Examples include 6-thio-7-deaza-2′-deoxyguanosine 5′-triphosphate (TDGTP) and 6-methoxy-7-deaza-2′-deoxyguanosine 5′-triphosphate (OMDG-TP), as well as 2′,3′-dideoxyguanosine 5′-triphosphate, 6-thio-2′-deoxyguanosine 5′-triphosphate (T-dGTP), D-carbocyclic-2′-deoxyguanosine 5′-triphosphate (D-CdG-TP), and 2',3'-dideoxythymidine 5'-triphosphate(ddTTP) [505, 506]. However, their efficacy still requires validation through both in vitro and in vivo experiments.

#### G-quadruplex stabilisers

Telomeres contain G-quadruplex secondary structures [507]. Telomeric G-quadruplexes are resolved by DNA helicases prior to telomere extension [508]. Compounds that stabilize G-quadruplexes can prevent telomerase from acting on the telomeres, thereby triggering a DNA damage response and cell death [509, 510]. G-quadruplex stabilizers are predominantly planar aromatic molecules, which primarily interact with G-quadruplex structures through π–π stacking and electrostatic interactions. In their design, it is imperative to balance robust binding affinity for G-quadruplexes with high selectivity for four‐stranded DNA and dsDNA [396]. G-quadruplex stabilizers are predominantly characterized by large, planar aromatic systems, yet their limited aqueous solubility poses a significant challenge. This limitation can be mitigated through structural modifications, such as the incorporation of protonatable side chains to enhance hydrophilicity while preserving the integrity of the hydrophobic core. Meanwhile, metal–organic complexes, distinguished by their structural and electronic versatility, have emerged in recent years as highly effective and increasingly predominant G4 ligands.

Quarfloxin, a fluoroquinolone derivative, can stabilize G4 structures in ribosomal DNA (rDNA) quadruplexes, thereby disrupting the interaction with nucleolin, inhibiting Pol I transcription, and triggering apoptotic cell death in cancer cells. It was the first G4 stabilizer to enter clinical trials [511, 512]. In Phase II clinical trials, quarfloxin demonstrated significant biological activity and good tolerability with a manageable toxicity profile; however, its pharmacokinetic properties led to the discontinuation of further research [512, 513]. CX-5461, another quarfloxin derivative, exhibited antitumor activity in animal models of pancreatic cancer and melanoma and showed favorable pharmacokinetics and moderate activity in Phase I clinical trials [514, 515]. However, it induced widespread mutations across different cell lines, even exceeding those caused by environmental carcinogens, which hindered its further clinical development [516].

Naphthalenediimide (NDI), through its aromatic core modified by various pendant groups, exhibits affinity for diverse G-quadruplex-forming sequences, enhancing the possibility of improved G-quadruplex binding selectivity and potency [517, 518]. The Neidle group conducted a series of studies on NDI derivatives and reported on the research of four distinct derivatives [519–522]. The first compound, BMSG-SH-3, when administered intraperitoneally in a mouse xenograft model using MIA-Pa-Ca2 cells, was found to rapidly enter the nucleus. This resulted in a 50% reduction in both tumor volume and TA, as well as a 30% decrease in the expression levels of HSP90 and hTERT proteins. However, due to deviations from Lipinski’s rule, primarily because of its molecular weight, the Neidle group initiated efforts to develop alternatives to BMSG-SH-3. The team subsequently named these compounds MM41, CM03, and SOP1812. Among them, SOP1812 exhibited strong affinity for hTERT G4 and HuTel21 G4, demonstrating the most potent inhibitory effects on pancreatic cancer by downregulating multiple dysregulated genes [271–276]. Due to its relatively superior biological activity, SOP1812 advanced to Phase I clinical trials for solid tumors, including pancreatic cancer [523].

PhenDC3, a phenanthroline-based ligand, exhibits strong π-π interactions with a G-quartet and can inhibit the unwinding of a unimolecular G4 substrate by FANCJ and DinG helicases [524, 525]. APTO-253, a derivative of PhenDC3, is a MYC inhibitor that does not induce bone marrow suppression, making it particularly suitable for the treatment of acute myeloid leukemia [526]. Its anticancer mechanism involves targeting G4 DNA, which leading to cell cycle arrest, DNA damage, and activation of the tumor suppressor Krüppel-like factor 4 (KLF4) [527]. Unfortunately, despite entering Phase I clinical trials, the development of this drug was halted [526]. TGP18 is another phenanthroline derivative that binds to the BCL-2 G4 with submicromolar affinity, thereby reducing BCL-2 mRNA levels in tumor cells and inducing apoptosis. In xenograft models of breast and lung cancers, it exhibited a tumor growth inhibition rate of approximately 60%, indicating promising potential for further research [528].

BRACO-19 is a 3,6,9-trisubstituted acridine derivative that binds to human DNA G-quadruplexes, inhibiting TA during the elongation of single-stranded telomere overhangs [529]. In cellular experiments, BRACO-19 significantly reduced hTERT expression in uterine cancer cells and TA in ovarian cancer cells [530, 531]. In animal models, intravenous administration led to the regression of early-stage transplanted tumors, whereas oral administration was ineffective [531]. However, its limited bioavailability and narrow therapeutic window have hindered further development [531, 532]. Its derivative, AS1410, has a longer half-life but showed limited activity in transplant tumor models and had insufficiently high tolerable doses, preventing further progression of related studies.

Kuang G et al. conducted a study on a series of newly synthesized bisacridine derivatives and identified compound a9 as capable of stabilizing both G-quadruplex and i-motif structures, thereby downregulating c-myc gene transcription [533]. This mechanism inhibits the proliferation of SiHa cells, induces apoptosis and cell cycle arrest, and reduces the volume of tumors in xenograft models by 41.6%, making it the most promising compound among those studied [533].

The pentacyclic acridine derivative RHPS4 inhibits the catalytic and capping functions of telomerase, causing the displacement of TERT from the nucleus, suppressing overexpressed TRF2 or POT1, and inducing DNA damage, thereby reducing telomere length and inducing senescence in MCF-7 breast cancer cells in vitro [534–536]. Among various tumor xenografts, RHPS4 demonstrates the strongest effect on CG5 breast cancer xenografts, achieving an inhibition rate of 80% and even a cure rate of 40%, with sustained therapeutic effects lasting for 30 days [537]. However, the cardiotoxic effects of RHPS4 in guinea pigs have limited its clinical investigation [538].

SYUIQ-5 is an indoloquinoline that, in vitro studies with Burkitt lymphoma cells, inhibits c-MYC transcription by stabilizing c-MYC G-quadruplexes and disrupting the binding of the protein NM23-H2 to DNA [539]. In xenograft tumor models, it reduces tumor volume by 27.4%. Another derivative, a triazole-containing benzofuran quinoline, exhibits a tumor growth inhibition rate of approximately 38% through a similar mechanism [539, 540].

Human topoisomerase I inhibitors, such as indotecan (NCT01051635) and indimitecan (NCT01051635), along with another compound (NCT03030417), have entered Phase I clinical trials [541]. These agents induce DNA damage by binding to c-MYC G-quadruplexes, leading to the downregulation of c-MYC. Such studies validate the clinical potential of this class of drugs [542].

Jinglin J et al. synthesized a new series of quindoline derivatives [543]. Screening revealed that the introduction of electron-donating groups, such as substituted amino groups at the C-11 position of the quindoline moiety, significantly enhanced the inhibitory effect on TA [543]. These compounds induce G-rich telomeric repeat sequences to fold into quadruplexes and stabilize the G-quadruplex structure, thereby inhibiting telomerase function [543].

Lu et al. designed 5-N-methyl quindoline (cryptolepine) derivatives, which carry a positive charge at the 5-N position of the aromatic quinoline scaffold [544]. This facilitates better binding to G-quadruplexes and inhibits TA, leading to the stagnation of cancer cell population growth. Subsequently, Xu W from the same team published research on 5-methyl-11-(2-morpholinoethylamino)-10H-indolo [3,2-b]quinolin-5-ium iodide (compound 1), demonstrating the potential of quindoline derivatives as anticancer agents [545].

Xiong et al. synthesized two chiral RuII-PtII dinuclear complexes, designated as Δ-RuPt and Λ-RuPt, which were encapsulated within biotin-functionalized DNA cages, thus named Δ-RuPt@biotin-DNA cage and Λ-RuPt@biotin-DNA cage, respectively [546]. Numerous reports have indicated that G-quadruplex stabilizers are often evaluated under nonphysiological and dilute conditions [547], which do not mimic the highly concentrated intracellular environment of up to 400 mg/mL of biomolecules in the nucleus [8]. Such conditions can significantly affect the stability of G-quadruplexes [548]. Consequently, to better adapt to the molecularly crowded intracellular milieu and bind to G-quadruplexes, these complexes were designed [546]. However, these agents exhibited high toxicity in various cancer cell lines but showed lower toxicity in HCC cells [546]. Administration of the two drugs to mice bearing cisplatin-resistant lung tumors via intravenous injection demonstrated a robust inhibition of tumor growth, highlighting the therapeutic potential of these agents [546].

### Treatment, cancer, and aging

Oncogenes are capable of inducing senescence, a process that differs from normal biological aging [549]. This observation underscores the paradoxical role of cellular senescence in the development and progression of cancer, wherein it can act both as a barrier to tumorigenesis and as a pro-oncogenic factor [550]. Animal experiments have even shown that stimulated senescent mouse embryonic fibroblasts may elicit an immune response within the host that promotes antitumor immunity mediated by dendritic cells (DCs) and CD8 + T cells [549]. Further research into biomarkers is necessary to discern those indicative of senescence processes that inhibit cancer from those that promote cancer [550]. Given the heterogeneity of the secretome produced by senescent cells, it may be necessary to develop targeted therapies tailored to the specific characteristics of different patients and cancer types [550]. Survivors of childhood cancers may experience chronic inflammation due to therapy-induced senescence of normal cells, resulting in relatively shorter telomeres compared with age-matched individuals without a history of cancer. Consequently, these survivors may manifest age-related chronic diseases or functional impairments, such as increased cardiovascular risk and cognitive decline, approximately two decades earlier than their counterparts without a history of the disease [551]. Therefore, in therapeutic strategies targeting telomeres and telomerase, it is essential to consider whether these interventions might exacerbate the consequences associated with cellular senescence. The age of the patient and the growth status of the tumor must be carefully evaluated [550]. Additionally, the impact of cellular senescence on both the tumor and the overall health of the patient during treatment should be thoroughly assessed to mitigate the adverse effects induced by drug-induced senescence [550]. From another perspective, certain TERT activator compounds (TACs) may prove beneficial in this context. Studies have indicated that these agents are well-tolerated and lack carcinogenic potential, potentially reducing the incidence of cancer through their anti-aging effects [552, 553]. Examples include the natural compound TA-65, the hormonal agent danazol, and the TERC-stabilizing compound BCH001 [554–556]. However, despite their potential anticancer properties, these agents may also exacerbate cancer progression in clinical settings. For instance, telomerase activators derived from purified astragalus root, such as TA-65, have been shown to improve longevity metrics, including glucose tolerance, osteoporosis, and skin health, in telomerase-deficient mice, without significantly elevating cancer incidence [554]. Experimental findings indicate a tenfold increase in telomerase RNA levels in the liver of treated mice [554]. However, TA-65 has also been observed to activate telomerase in HCC cells, potentially influencing cellular growth and metastatic behavior [557]. Therefore, for patients already diagnosed with cancer, the application of TACs may require further investigation, given the potential impairment of cellular checkpoint functions [558]. Simultaneously, it is essential to investigate whether telomerase activators may accelerate cancer progression in cancers with high telomerase expression. If these activators do not promote cancer cell proliferation in patients, they may help sustain the vitality of other cells, thereby countering cancer or mitigating the adverse effects of cancer treatments [559].

## Conclusions and perspectives

The presence of telomeres and the enzyme activity of telomerase, on the surface, are proteins that resist genomic instability, thereby playing a key role in avoiding cancer by maintaining chromosome integrity. Their basic function is to ensure the integrity of genetic material during cell division. However, this protective mechanism appears to have a dual nature. Following various stimuli, telomeres and telomerase become pivotal factors in carcinogenesis, enabling cells to bypass cellular checkpoints and conferring upon cancer cells the capacity for unlimited proliferation and metastasis. At the same time, their survival also depends on the inactivation of cellular checkpoints. The misexpression or mutation of these entities is intricately involved in the entire continuum of cancer evolution, from the onset of precancerous lesions to the escalation into advanced malignancy. A synthesis of pertinent research findings indicates that when genes encoding components of the telomere machinery are underexpressed, leading to potential protein deficiencies, it may predispose cells to an increased susceptibility to cancer. Post-onset of cancer, genes associated with various parts of the telomere can exhibit overexpression, and the effects of this overexpression extend beyond merely enhancing TA. These components demonstrate extratelomeric functions within the biological system, suggesting roles in influencing cellular processes that are distinct from their conventional functions in telomere maintenance. Current research has revealed that the mechanisms underlying telomerase reactivation vary across different cell lines within the same type of cancer and among different cancer types. Even within the same reactivation mechanism, distinct pathways may be involved. However, the reasons behind these variations remain inadequately understood.Moreover, prior to the onset of cancer, the components of the telomerase complex often exhibit significant abnormalitie, typically manifesting as overexpression. This overexpression promotes the reactivation of telomerase, helps maintain telomere length, and assists in the continuous proliferation of cancer cells. We may be able to stratify the occurrence and development of cancer based on telomere shortening and elongation, as well as the levels of TA.

With the deepening of our understanding of telomeres, telomerase, and their related biological mechanisms, numerous biomarkers and treatment methods have begun to be studied. When used as biomarkers, telomeres and telomerase in conjunction may provide more accurate results. The mechanisms behind some contradictory study findings need further exploration. For example, different biomarkers have varying levels at the subcellular level, which may make the meaning represented by each biomarker more accurate. Contradictory results among biomarkers across different studies might be reasonably explained through subcellular statistical analysis. Notably, TL is influenced by factors such as ethnicity and age, leading to a lack of standardization in defining normal or abnormal TL thresholds. This variability in defining TL thresholds can introduce challenges in interpreting results and making comparisons across studies. Standardization of TL measurement methodologies and criteria for defining TL abnormalities will enhance the reliability and comparability of findings in future research. Moreover, current cohort studies are constrained by limitations related to geographic regions, ethnic populations, and inconsistencies in detection standards, which may contribute to the observed discrepancies in conclusions. Addressing these challenges will likely require collaboration among hospitals and research institutions across multiple regions. This effort should encompass both comprehensive, global statistical analyses to characterize the features of the human telomerase system and region- and ethnicity-specific stratifications based on genetic backgrounds. Such an approach would provide deeper insights into the roles of telomeres and telomerase in cancer. Additionally, although telomerase offers certain advantages in distinguishing tumor malignancy, predicting prognosis, and complementing morphological examination, its practical application remains limited. However, this situation may improve with advances in detection methods. Cancer screening may lead to resource waste due to the low incidence of cancer in the target population [560]. Utilizing easily detectable methods for assessing telomeres and TA through non-invasive means such as stool or blood samples as a pre-screening condition may help clinicians identify high-risk populations. This approach may reduce unnecessary harm and costs to patients, and by facilitating early detection and treatment, it may save healthcare resources and reduce patient suffering.

Therefore, when inhibiting these proteins in cancer cells, it is important to consider whether normal cells might also be affected in terms of their telomeres or telomerase components, potentially leading to adverse systemic effects, even though TA is typically negative in normal cells. Current therapeutic approaches targeting telomerase still exhibit limitations in clinical application, including notable side effects and variable efficacy across different individuals. Further research is needed to elucidate the underlying mechanisms and improve these treatments. If combined with drugs that address these side effects, it may be possible to increase the doses and frequency of immunotherapies and telomerase inhibitors, thereby enhancing their efficacy in treating cancer while avoiding patient intolerance. A deeper understanding of these processes will not only help promote the development of more precise cancer treatments but also shed light on the profound connections between cancer and the aging process, thus enriching our knowledge foundation in these critical areas of biomedicine. At present, research on telomerase inhibitors is primarily confined to cellular and animal models. While these studies have provided innovative insights into cancer treatment, they also exhibit certain limitations. For instance, in cellular experiments, the efficacy of a single telomerase inhibitor may vary significantly not only across different types of cancer cells but also among distinct cell lines of the same cancer type. The underlying mechanisms for these variations remain unclear and warrant further investigation. Such studies could pave the way for the development of telomerase inhibitors tailored to specific cancer types, as well as broad-spectrum inhibitors applicable to multiple cancer types. From the perspective of animal studies, differences between human and murine telomerase systems present additional challenges. Designing mouse models that more accurately mimic the physiological conditions of cancer patients requires further exploration. Achieving this goal necessitates a deeper understanding of the relationship between human telomerase and cancer, alongside advanced genetic engineering to optimize and humanize the telomerase system in mice. These efforts are critical for enhancing the translational relevance of preclinical findings and advancing therapeutic strategies.

## Data Availability

No datasets were generated or analysed during the current study.

## Abbreviations

TA: Telomerase activity
ALT: Alternative lengthening of telomeres
TRF1: Telomeric repeat-binding factors
POT1: Protection of telomeres 1
TIN2: TRF1-interacting protein 2
TPP1: POT1-TIN2 organizing protein
TERC: Telomerase RNA component
RAP1: Repressor/activator protein 1
TER: Telomerase RNA
TERT: Telomerase reverse transcriptase
TRBD: Telomerase Essential N-terminal
hTERT: Human telomerase reverse transcriptase
RT: Reverse transcriptase
CTE: C-Terminal extension
HGPIN: High-grade prostatic intraepithelial neoplasia
IPMN: Intraductal papillary mucinous neoplasm
HCC: Hepatocellular carcinoma
LGDN: Low-grade dysplastic neuroepithelial
HGDN: High-grade dysplastic neuroepithelial
LGD: Low-grade dysplasia
HGD: High-grade dysplasia
TPE-OLD: Telomere position effects over long distances
MDSCs: Myeloid‐derived suppressor cells
mtDNA: Mitochondria and the mitochondrial genome
Nek7: NIMA-related kinases 7
Sp1: Specificity protein 1
NSCLC: Non-small cell lung cancer
TERRA: Telomeric repeat-containing RNA
BCL-2: B-cell lymphoma 2
CIN: Cervical intraepithelial neoplasia
OB: Oligosaccharide-binding
THOR: TERT hypermethylated oncological region
TSS: Transcription start site
ETS: E-twenty-six
TL: Telomere length
RIOK2: Ribosomal Protein S6 Kinase 2
DKC1: Dyskerin
lncRNAs: Long noncoding RNAs
mRNAs: Messenger RNAs
mtDNA: Mitochondrial DNA
NPC: Nasopharyngeal carcinoma
CCL2: Chemokine (C–C motif) ligand 2
TLV: Telomere length variation
CAF: Cancer-associated fibroblast
LTL: Leukocyte telomere length
cfDNA: Cell-free DNA
OS: Overall survival
PFS: Progression-free survival
NCHCC: Non-clear cell hepatocellular carcinoma
CCHCC: Clear cell hepatocellular carcinoma
LA: Luminal A
LB: Luminal B
TN: Triple-negative
PST: Primary systemic therapy
GIC: Gastrointestinal Cancer
ctDNA: Circulating tumor DNA
ER: Estrogen receptor
PR: Progesterone receptor
HER2: Human epidermal growth factor receptor 2
ILC: Invasive lobular carcinoma
IDC: Invasive ductal carcinoma
TNBC: Triple-negative breast cancer
PSC: Pulmonary sarcomatoid carcinoma
AL: Acute leukemia
AML: Acute myeloid leukemia
ALL: Acute lymphoblastic leukemia
APL: Acute promyelocytic leukemia
B-ALL: B-acute lymphoblastic leukemia
T-ALL: T-Acute Lymphoblastic Leukemia
BMMNC: Bone marrow mononuclear cells
CL: Chronic leukemia
CLL: Chronic lymphocytic leukemia
CML: Chronic myeloid leukemia
CP: Chronic phase
AP: Accelerated phase
BP: Blast crisis phase
MIS: Melanoma in situ
NM: Nodular melanoma
SSM: Superficial spreading melanoma
LMM: Lentigo maligna melanoma
UM: Uveal melanoma
ALM: Acral lentiginous melanoma
MM: Mucosal melanoma
AM: Acral melanoma
UV: Ultraviolet
EBF1: Early B-cell factor 1
PRC2: Polycomb repressive complex 2
HDACs: Histone deacetylases
ITGB1: Integrin β1
mtDNA: Mitochondrial DNA
ECM: Extracellular matrix
thyrocytes: Follicular thyroid cells
PTC: Papillary thyroid carcinoma
FTC: Follicular thyroid carcinoma
PDTC: Poorly differentiated thyroid carcinoma
ATC: Anaplastic thyroid carcinoma
UTSS: Upstream of the transcription start site
HBV: Hepatitis B
HCV: Hepatitis V
ICC: Intrahepatic Cholangiocarcinoma
cHCC-ICC: Combined hepatocellular-cIntrahepatic cholangiocarcinoma
AR: Androgen receptor
UCs: Urothelial carcinomas
BLCA: Bladder urothelial carcinoma
CpG-ODN: CpG oligodeoxynucleotide
PDC: Plasmacytoid dendritic cells
CTL: Cytotoxic T lymphocyte
CNS: Central nervous system
PBMC: Peripheral blood mononuclear cell
JNK: C-Jun N-terminal kinase
EGCG: Epigallocatechin gallate
MMP: Matrix metalloproteinases
OSE: Ovarian surface epithelial
AFP: Alpha-fetoprotein
GSK-3β: Glycogen synthase kinase-3β
HIV: Human immunodeficiency virus
rDNA: Ribosomal DNA
TACs: TERT activator compounds
